# F-Box Protein Specificity for G1 Cyclins Is Dictated by Subcellular Localization

**DOI:** 10.1371/journal.pgen.1002851

**Published:** 2012-07-26

**Authors:** Benjamin D. Landry, John P. Doyle, David P. Toczyski, Jennifer A. Benanti

**Affiliations:** 1 Program in Gene Function and Expression, University of Massachusetts Medical School, Worcester, Massachusetts, United States of America; 2 Program in Molecular Medicine, University of Massachusetts Medical School, Worcester, Massachusetts, United States of America; 3 Department of Biochemistry and Biophysics, University of California San Francisco, San Francisco, California, United States of America; The University of North Carolina at Chapel Hill, United States of America

## Abstract

Levels of G1 cyclins fluctuate in response to environmental cues and couple mitotic signaling to cell cycle entry. The G1 cyclin Cln3 is a key regulator of cell size and cell cycle entry in budding yeast. Cln3 degradation is essential for proper cell cycle control; however, the mechanisms that control Cln3 degradation are largely unknown. Here we show that two SCF ubiquitin ligases, SCF^Cdc4^ and SCF^Grr1^, redundantly target Cln3 for degradation. While the F-box proteins (FBPs) Cdc4 and Grr1 were previously thought to target non-overlapping sets of substrates, we find that Cdc4 and Grr1 each bind to all 3 G1 cyclins in cell extracts, yet only Cln3 is redundantly targeted *in vivo*, due in part to its nuclear localization. The related cyclin Cln2 is cytoplasmic and exclusively targeted by Grr1. However, Cdc4 can interact with Cdk-phosphorylated Cln2 and target it for degradation when cytoplasmic Cdc4 localization is forced *in vivo*. These findings suggest that Cdc4 and Grr1 may share additional redundant targets and, consistent with this possibility, *grr1Δ cdc4-1* cells demonstrate a *CLN3*-independent synergistic growth defect. Our findings demonstrate that structurally distinct FBPs are capable of interacting with some of the same substrates; however, *in vivo* specificity is achieved in part by subcellular localization. Additionally, the FBPs Cdc4 and Grr1 are partially redundant for proliferation and viability, likely sharing additional redundant substrates whose degradation is important for cell cycle progression.

## Author Summary

Most cells only divide when they receive the proper cues. When a cell receives a signal to divide, levels of G1 cyclin proteins increase and drive entry into the cell division cycle. Overexpression of G1 cyclins can drive cells into the cell cycle inappropriately and thus may contribute to the hyperproliferation of cancer cells. Despite the importance of controlling G1 cyclin levels, the mechanisms regulating the degradation of these proteins are not well understood. We have now elucidated the mechanism of degradation of the yeast G1 cyclin Cln3. In contrast to related cyclins in yeast, Cln3 is targeted for degradation by two redundant pathways, which act to keep Cln3 levels extremely low. This finding may have implications for understanding how G1 cyclins are degraded in human cells and how expression of G1 cyclins may be misregulated during cancer development.

## Introduction

The ubiquitin-proteasome system plays an essential role in controlling passage through the eukaryotic cell cycle [1]. A significant fraction of cell cycle-regulated ubiquitination is carried out by SCF (Skp1-Cullin-F-box protein) family ubiquitin ligases, which target numerous cell cycle regulators for proteasomal degradation. All SCF ligases consist of three core subunits: a structural cullin subunit (Cdc53 in yeast, Cul1 in mammals), an adaptor protein (Skp1) and a RING finger protein (Rbx1), plus one of a family of modular substrate-specificity subunits called F-box proteins (FBPs) [2]–[7]. There are large numbers of FBPs in all eukaryotes, and each is believed to target the SCF to a specific set of substrates by interacting with distinct epitopes in those proteins. In almost all instances, FBPs recognize proteins that have been post-translationally modified, usually by phosphorylation, which enables ubiquitination to be regulated by substrate modification [8].

In budding yeast, the FBPs Cdc4 and Grr1 have well-established cell cycle-regulatory roles [1]. Both FBPs recognize phosphorylated epitopes in their substrates, however they bind to these epitopes through distinct phosphorecognition domains: a WD40 repeat domain in Cdc4 and a leucine rich repeat domain in Grr1 [8]. Interestingly, although Grr1 and Cdc4 are thought to have entirely non-overlapping sets of substrates, each is capable of interacting with targets that have been phosphorylated by cyclin dependent kinase (Cdk). This group of substrates includes several proteins that regulate entry into S phase including the Grr1 substrates Cln1 and Cln2 [9], as well as the Cdc4 substrates Sic1 [10] and Cdc6 [11]. In addition to this group of defined SCF targets, Cdk phosphorylates hundreds of yeast proteins [12], [13], and many of these are rapidly degraded [14], suggesting that there is a widespread connection between Cdk phosphorylation and protein degradation. However, the majority of these proteins have not been identified in genome-wide screens for Cdc4 or Grr1 targets [15], [16], suggesting that they may be targeted for degradation by alternate ubiquitin ligases.

One such Cdk-phosphorylated protein is the G1 cyclin Cln3. Similar to cyclin D1 in mammals, Cln3 is the furthest upstream cyclin, which senses growth cues and triggers entry into the cell cycle. Cells become committed to progress through the cell cycle upon phosphorylation of the transcriptional repressor protein Whi5 by Cln3/Cdc28, which leads to Whi5 inactivation and increased expression of downstream genes including the related cyclins Cln1 and Cln2 [17], [18]. Consistent with Cln3 having a critical role in cell cycle entry, its levels are very tightly controlled. In addition to being regulated by transcription [19], [20] and subcellular localization [21]–[23], Cln3 is rapidly degraded. This proteolytic degradation is critical to restrain Cln3 activity, since expression of a truncated and stable form of the Cln3 protein drives cells through G1 phase prematurely, resulting in a significant reduction in cell size [24]–[26]. Despite the physiological importance of Cln3 degradation, the ubiquitin ligase that targets Cln3 for degradation has not been identified.

Previous studies have implicated an SCF ligase in Cln3 degradation [26], [27], however no FBP has been identified that recognizes Cln3. Here, we show that Cdc4 and Grr1 redundantly target Cdk-phosphorylated Cln3 for degradation. Mutation of either FBP alone has no detectable effect on Cln3 levels or stability, yet Cln3 is completely stable in double mutant cells. Surprisingly, we find that both Cdc4 and Grr1 interact with all 3 G1 cyclins (Cln1, Cln2 and Cln3) in cell extracts, however only Cln3 is redundantly targeted *in vivo*, because it is the only G1 cyclin that localizes primarily to the nucleus. Cln2 is cytoplasmic and exclusively targeted by Grr1 [9], [28]. However, we show that Cdc4 can target Cln2 for degradation when cytoplasmic Cdc4 localization is forced *in vivo*. Finally, we observed a synthetic growth defect in *cdc4 grr1* double mutant cells that is not suppressed by deletion of *CLN3*. In sum, these data demonstrate that the binding specificities of FBPs do not necessarily dictate which proteins are *in vivo* targets, and suggest that Cdc4 and Grr1 have additional redundant targets whose regulated degradation is necessary for normal cell cycle control.

## Results

### SCF^Grr1^ and SCF^Cdc4^ Redundantly Target Cln3 for Degradation

**Figure 1:**
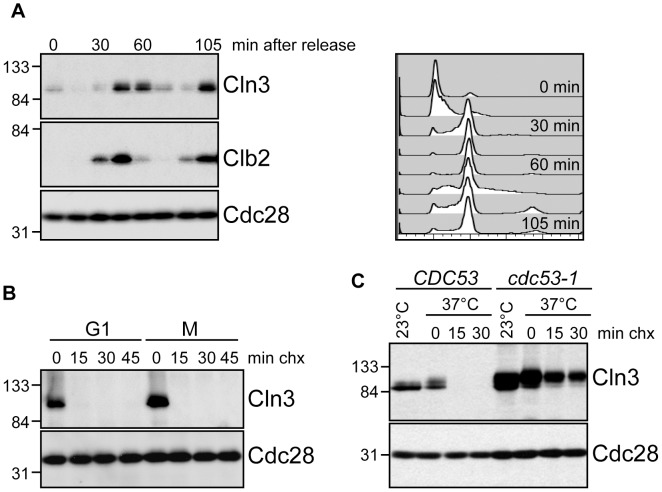
Cell cycle regulation of Cln3. (A) Cln3 levels peak in mitosis. Cells expressing Cln3-13Myc were synchronized in G1 with alpha-factor and released into the cell cycle. Samples were collected for flow cytometry and Western blot analysis at 15-minute intervals. Western blots for Cln3-13Myc, Clb2 and Cdc28 are shown. Cell cycle position was monitored by flow cytometry, right. Quantification of Cln3 protein levels is shown in Figure S1A. (B) Cln3 is unstable in G1 and mitosis. Cycloheximide-chase assay showing levels of Cln3-13Myc and Cdc28 in cells arrested in G1 with alpha-factor, or in mitosis (M) with nocodazole, and then treated with cycloheximide for the indicated number of minutes (min chx). Cell cycle profiles are shown in Figure S1B. (C) Cln3 stability is regulated by Cdc53. Cycloheximide-chase assay showing levels of Cln3-13Myc and Cdc28 in wild-type cells (*CDC53*) and *cdc53-1* temperature-sensitive cells before (23°C) and after (37°C) incubation at the non-permissive temperature. Following incubation at 37°C, cycloheximide was added for the indicated number of minutes. Cell cycle profiles are shown in Figure S1C. For all gels, molecular weight markers are indicated at the left.

To better understand the regulation of Cln3 degradation, we examined Cln3 protein levels throughout the cell cycle and found that they paralleled the reported transcriptional expression profiles [19], [20], [29], rising in mitosis, shortly after the mitotic cyclin Clb2, and persisting through G1 phase (Figure 1A; Figure S1A). Cln3 was rapidly degraded in cells arrested in either G1 or mitosis, demonstrating that it is degraded throughout the cell cycle (Figure 1B; Figure S1B). Previous data suggested that an SCF ubiquitin ligase is responsible for targeting Cln3 [26], [27]. Consistent with these observations, we found that phosphorylated Cln3 is stable and accumulates in cells expressing a temperature-sensitive allele of *CDC53* (*cdc53-1*) upon shift to the restrictive temperature (Figure 1C; Figure S1C). Similarly, transcriptional shut-off of the genes encoding Cdc53 or Cdc34 (the E2 enzyme that functions with SCF ligases) stabilized Cln3 (Figure S1D).

**Figure 2:**
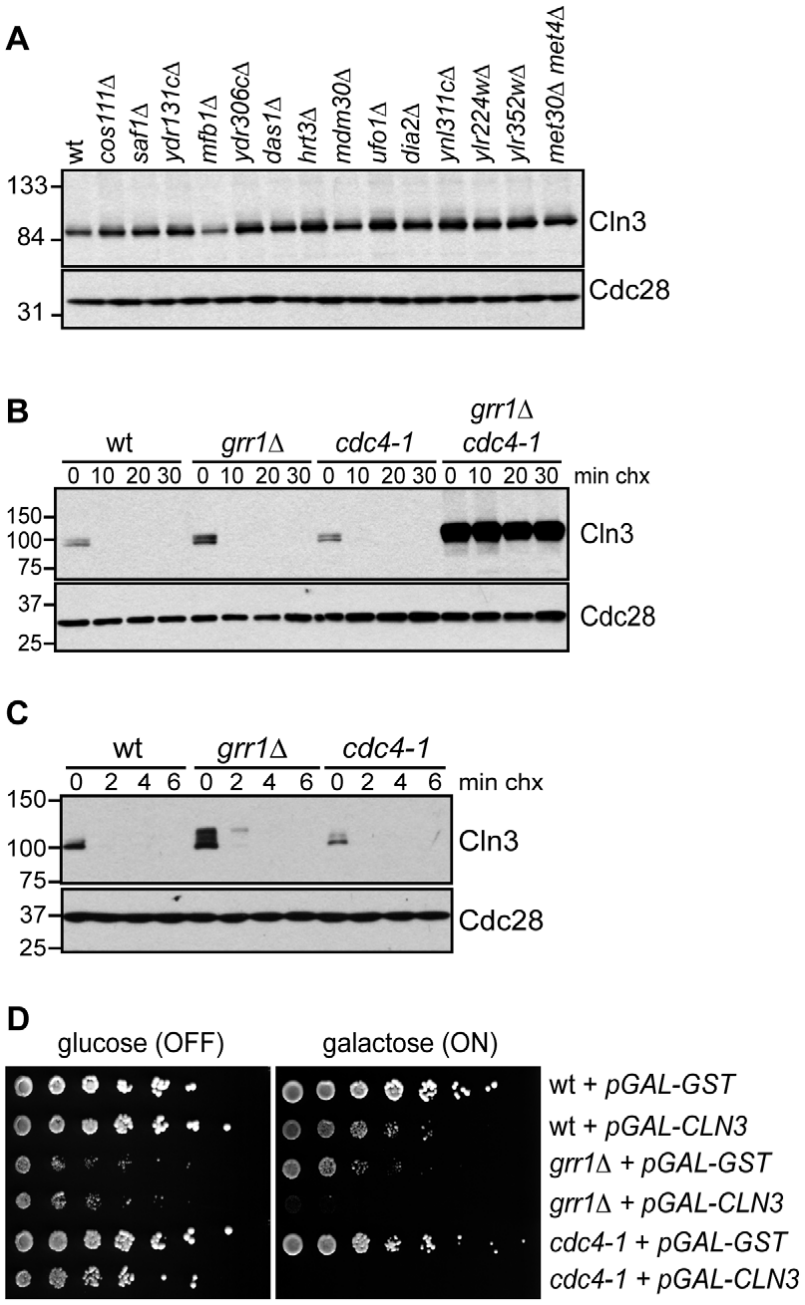
Cln3 degradation is redundantly regulated by Cdc4 and Grr1. (A) Cln3 levels are not regulated by any single F-box protein. Western blot of Cln3-13Myc in wild-type cells (wt) and cells deleted for individual F-box protein genes, as indicated. All cells were arrested in mitosis, to control for differences in cell cycle position. The F-box protein Met30 is essential, so Cln3-13Myc was examined in cells lacking both Met30 and its target Met4, which renders Met30 nonessential. (B) Cln3 is stable in *grr1Δ cdc4-1* cells. Cycloheximide-chase assay showing levels of Cln3-13Myc in wild-type (wt), *grr1Δ*, *cdc4-1*, and *grr1Δ cdc4-1* cells after shifting to the non-permissive temperature for 2 hours, followed by treatment with cycloheximide for the indicated number of minutes (min chx). Levels of Cln3-13Myc and Cdc28 are shown. (C) Cln3 is unstable in *grr1Δ* and *cdc4-1* single mutant strains. Cycloheximide-chase assay as in (B) except that cells were collected at 2 minute intervals. For all gels, molecular weight markers are indicated at the left. (D) Cells with the indicated genotypes were transformed with a high-copy plasmid expressing *CLN3* from a galactose inducible promoter (*pGAL-CLN3*) or a control high-copy plasmid expressing GST (*pGAL-GST*). 5-fold dilutions of cells were plated on glucose (*CLN3* transcription OFF) and galactose (*CLN3* transcription ON) and incubated at 23°C (the permissive temperature for *cdc4-1*).

We next attempted to identify the specific FBP that targets Cln3 for degradation. Cln3 levels were compared among strains carrying single deletions of every non-essential yeast F-box protein (Figure 2A; data not shown), however no single deletion caused a significant increase in Cln3 protein levels. Furthermore, inactivation of the essential FBP Cdc4 did not stabilize Cln3 (Figure 2B; Figure S1D). Together, these data suggested that Cln3 might be redundantly targeted by two or more FBPs. The most likely candidate FBPs to redundantly regulate Cln3 were Grr1 and Cdc4, since each of these proteins is known to target Cdk-phosphorylated substrates [9]–[11], [30], [31]. Indeed, we found that simultaneous inactivation of Grr1 and Cdc4 led to complete stabilization of Cln3, whereas deletion of *GRR1* or inactivation of Cdc4 alone had no significant effect on Cln3 levels or stability (Figure 2B). Cln3 protein was almost undetectable in both single mutant strains after only 2 minutes of cycloheximide treatment (Figure 2C), and reintroduction of either Cdc4 or Grr1 into *grr1Δ cdc4-1* cells led to an equivalent reduction of Cln3 levels (Figure S2A). Consistent with these observations, overexpression of Cln3 in either *grr1Δ* cells, or cells with limiting amounts of Cdc4 (*cdc4-1* grown at the permissive temperature) was lethal (Figure 2D), indicating that both FBPs are required for the cell to tolerate high levels of Cln3. Together, these data demonstrate that Cln3 is redundantly regulated by Cdc4 and Grr1.

### Cdc4 and Grr1 Interact with Cdk-Phosphorylated Cln3

**Figure 3:**
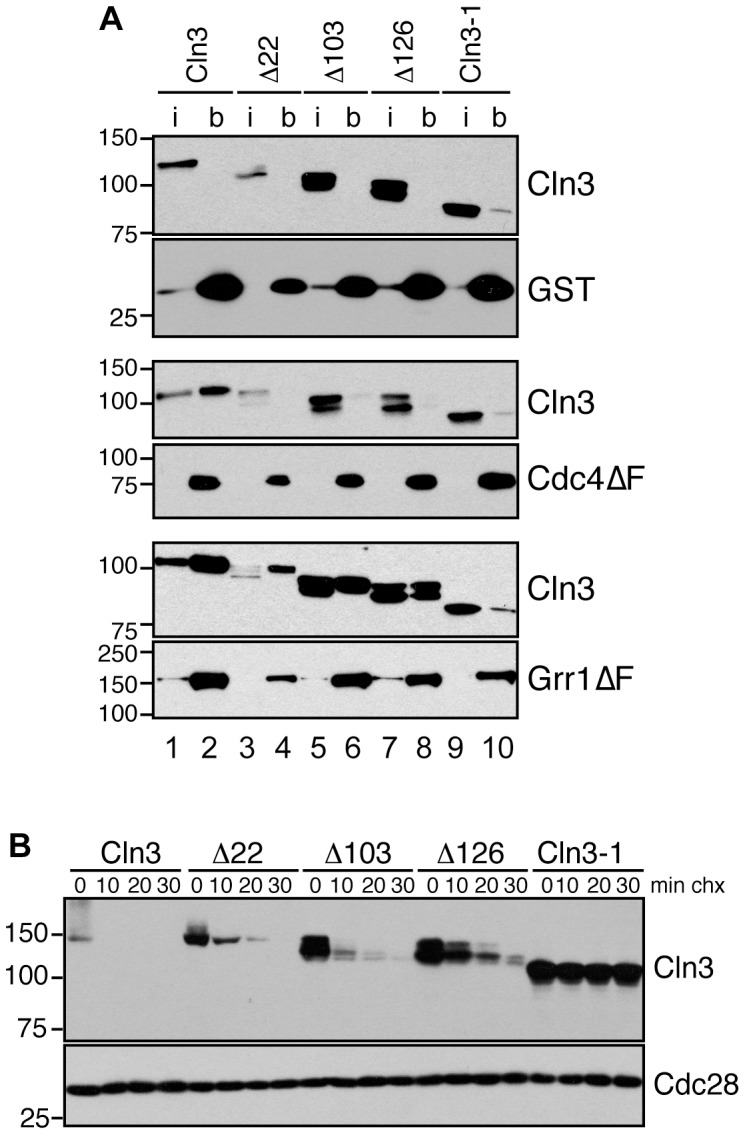
Cln3 interacts with Cdc4 and Grr1. (A) Cln3 interacts with Cdc4 and Grr1. Myc and GST Western blots showing pull-downs of GST, GST-Cdc4ΔF and GST-Grr1ΔF proteins from *grr1Δ cdc4-1* cells expressing Cln3-13Myc (wt) or Cln3 truncation mutants (described in Figure S2B). 2% input (i) and glutathione-sepharose bound proteins (b) are shown. (B) Cycloheximide-chase assay of Cln3 truncation mutants from (A) expressed in wild-type cells. Western blots for Myc and Cdc28 are shown. For all gels, molecular weight markers are indicated at the left.

In order to demonstrate that Cdc4 and Grr1 directly target Cln3, we examined the binding of Cln3 to each FBP. Because complexes between ubiquitin ligases and substrates are unstable and often difficult to detect, we assayed binding of Cln3 to GST-tagged Grr1 or Cdc4 proteins that lack the F-box domain and therefore cannot interact with the remainder of the SCF ubiquitin ligase complex (Cdc4ΔF and Grr1ΔF) [15], [32]. In contrast to the full-length proteins, expression of GST-Cdc4ΔF or GST-Grr1ΔF had no effect on levels of Cln3 (Figure S2A), confirming that they were not incorporated into active SCF complexes. Upon pull-down of GST-tagged proteins from cellular lysates, Cln3 associated with both Cdc4ΔF and Grr1ΔF proteins but not with GST (Figure 3A, lane 2), supporting the model Cdc4 and Grr1 directly target Cln3 *in vivo*.

Previous studies suggested that the C-terminal tail of Cln3 is required for its degradation [26] . To verify this result and narrow down the region required for Cln3 degradation we constructed a series of C-terminal truncation mutants (Figure S2B) and examined their half-lives *in vivo*. With the exception of the largest deletion of 177 amino acids (equivalent to the previously characterized allele Cln3-1; [26]), all mutants were still turned over at significant rates (Figure 3B). Although each of these mutants was slightly more stable than wild-type Cln3, cells expressing these mutants did not show any obvious changes in cell cycle progression (Figure S2E–S2F), suggesting that partial stabilization does not impact the cell cycle *in vivo*. However, since each truncated protein was more stable than wild-type Cln3 in either *grr1Δ* or *cdc4-1* cells (Figure 2B), this indicates that these mutations must partially interfere with degradation by both FBPs.

The large size of the C-terminal tail makes it possible that each FBP recognizes a distinct epitope within this domain. Therefore, to further narrow down the requirements for binding to each FBP, we analyzed the binding of the C-terminal Cln3 truncation mutants to Cdc4 and Grr1. Interestingly, we found that all truncations disrupted binding to Cdc4 (Figure 3A, middle panels), including the smallest deletion of just 22 amino acids. In contrast, all truncations except for the largest deletion (Cln3-1) bound well to Grr1, albeit at reduced levels (Figure 3A, bottom panels). These data suggest that while Grr1 and Cdc4 both require the Cln3 C-terminus for binding, they do not bind identical epitopes.

**Figure 4:**
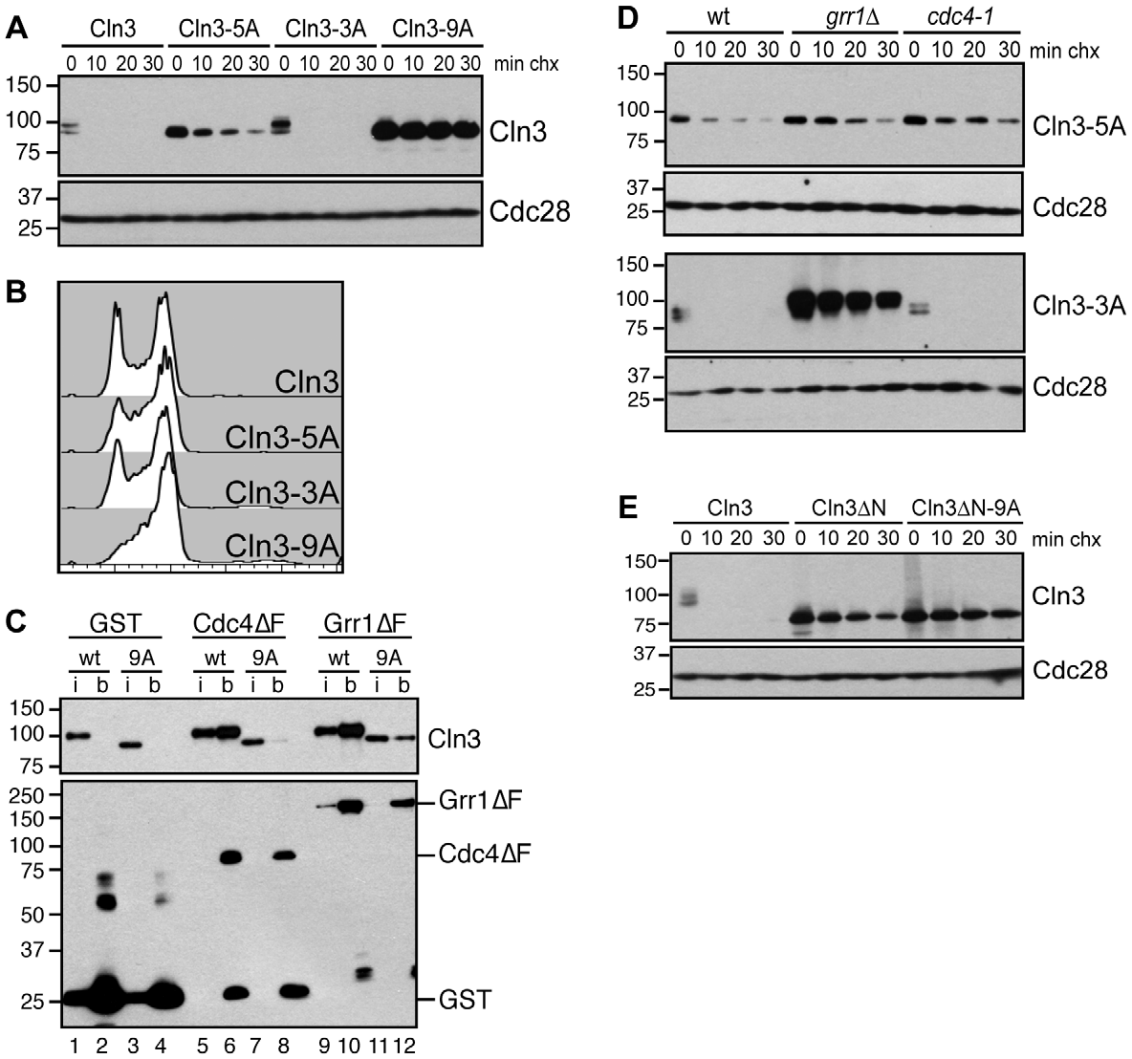
Cdk-phosphorylation of the Cln3 C-terminus is required for its degradation. (A) Mutation of Cdk consensus sites stabilizes Cln3. Cycloheximide-chase assay showing levels of Cln3-13Myc, Cln3-5A-13Myc, Cln3-3A-13Myc and Cln3-9A-13Myc after the addition of cycloheximide for the indicated number of minutes (min chx). Cdc28 is shown as a loading control. Diagram of mutant alleles is shown in Figure S2B. (B) Expression of Cln3-9A accelerates progression through G1 phase. Cell cycle profiles of asynchronous wild-type cells and cells expressing Cln3-3A, Cln3-5A, or Cln3-9A. (C) Cdc4 and Grr1 bind to Cdk-phosphorylated Cln3. Myc and GST Western blots showing pull-downs of GST, GST-Cdc4ΔF and GST-Grr1ΔF proteins from *grr1Δ cdc4-1* cells expressing Cln3-13Myc (wt) or Cln3-9A-13Myc (9A). 2% input (i) and glutathione-sepharose bound proteins (b) are shown. (D) Cdc4 and Grr1 require different regions of the Cln3 C-terminus for targeting. Cycloheximide-chase assays of Cln3-5A-13Myc (top panels) and Cln3-3A-13Myc (bottom panels) in wild-type (wt), *grr1Δ* or *cdc4-1* cells. Cells were shifted to 37°C for 2 hours and cycloheximide was added for the indicated number of minutes. Cln3-13Myc and Cdc28 Western blots are shown. (E) Cdk-phosphorylation of Cln3 occurs *in cis*. Cycloheximide-chase assay of full-length Cln3-13Myc, Cln3ΔN-13Myc (Cln3 lacking amino acids 2-207), and Cln3ΔN-9A-13Myc. Each protein is expressed from the *TEF1* promoter. For all gels, molecular weight markers are indicated at the left.

Cdk-phosphorylation of the Cln3 C-terminus is also required for its degradation [27], [33]. Since both Cdc4 and Grr1 bind Cdk-phosphorylated epitopes, this suggested that both FBPs might bind to the Cln3 C-terminus in a phospho-dependent manner. To test this, we first constructed a stable, Cdk-deficient allele of Cln3. The Cln3 C-terminus includes 10 serine/threonine-proline motifs, constituting the minimal Cdk-consensus site. A previous report demonstrated that mutation of the single full Cdk consensus site (S/TPxK) in the C-terminus of Cln3, serine 468, partially stabilized a Cln3 C-terminus-β-Gal fusion protein [27]. Notably, mutation of this site in the context of the full-length protein only had a minor effect on Cln3 stability and did not affect cell cycle position (Figure S2C–S2D). We then changed all of the remaining C-terminal Cdk consensus sites to alanine residues within the endogenous *CLN3* locus and assayed the stability of the mutated Cln3 proteins. Mutation of the 9 most C-terminal sites completely stabilized Cln3 (Figure 4A, Cln3-9A), and led to a cell cycle profile consistent with a significantly shortened G1 phase (Figure 4B). Next, we tested whether Cln3-9A could interact with Cdc4 and Grr1. Consistent with our prediction, significantly less Cln3-9A bound to each FBP, in comparison to wild-type Cln3 (Figure 4C, compare lanes 6 & 8, 10 & 12). These data strongly suggest that phosphorylation of the Cln3 C-terminus contributes to its interaction with both Cdc4 and Grr1.

Although Cdk phosphorylation of the C-terminus is required for Cln3 degradation, our binding data suggested that each FBP recognizes a distinct epitope within this domain, so we further analyzed the requirements for these C-terminal phosphosites in degradation by each FBP. Both Cdc4 and Grr1 are thought to target regions of their substrates that include multiple Cdk-phosphorylated residues [9]–[11], [30], [31], so we mutated two clusters of Cdk phosphosites in attempt to specifically interfere with binding to one or the other FBP (Figure S2B). First, we mutated five Cdk sites in the N-terminal half of the C-terminal domain, spanning residues 447–468 (designated Cln3-5A). Interestingly, we found that Cln3-5A was partially stabilized in wild-type cells (Figure 4A), and Cln3-5A-expressing cultures contained a slightly reduced fraction of cells in G1 phase (Figure 4B). Since degradation of wild-type Cln3 is unaffected in either *cdc4-1* or *grr1Δ* cells, the partial stabilization of the Cln3-5A protein suggested that these mutations partially interfered with targeting by both Cdc4 and Grr1. Consistent with this possibility, Cln3-5A was further stabilized in both *cdc4-1* and *grr1Δ* single mutant cells (Figure 4D, top panels).

Next, we mutated three sites at the extreme C-terminus (designated Cln3-3A). Notably, these sites are adjacent to the region required for Cdc4 interaction (Figure 3A), and are separated by two amino acids each, which matches the doubly phosphorylated degron motif that is preferred by Cdc4 [34], [35]. In wild-type cells Cln3-3A was rapidly degraded and its expression had no effect on cell cycle progression (Figure 4A–4B). However, Cln3-3A was degraded differently in cells lacking *GRR1* and *CDC4*: Cln3-3A turnover was unaffected in *cdc4-1* cells, but almost completely blocked in *grr1Δ* cells (Figure 4D, bottom panels). This demonstrates that Cln3-3A cannot be targeted by Cdc4 and is consistent with the prediction that degradation by Cdc4, but not Grr1, requires phosphorylation of these three C-terminal sites.

Together, these data suggest that although the Cdk-phosphorylated C-terminus of Cln3 is required for its targeting by Cdc4 and Grr1, the two FBPs recognize different epitopes within this domain. However, since Cln3 is thought to be constitutively bound to Cdk, it is possible that these sites are all constitutively phosphorylated *in cis* and that this phosphorylation allows Cln3 to be degraded by both FBPs throughout the cell cycle. A second possibility is that Cln3 is phosphorylated *in trans* by an alternate cyclin/Cdk complex in order to be targeted for degradation. Phosphorylation *in trans* could lead to cell cycle-regulated Cln3 degradation. To test whether *cis* phosphorylation of Cln3 by Cdk is required for its turnover, we constructed an N-terminal truncation mutant of Cln3 that removes the cyclin box and therefore can not bind to Cdk (Cln3ΔN). Interestingly, blocking *cis* phosphorylation in this manner led to almost complete stabilization of Cln3 protein (Figure 4E). The subtle degradation that was observed was dependent upon Cdk-phosphorylation, since mutation of the 9 C-terminal Cdk sites further stabilized the protein, suggesting that this protein could be phosphorylated by alternative cyclin/Cdk complexes. However, since Cln3ΔN is considerably more stable that full length Cln3, we conclude that the primary mode of Cln3 degradation depends upon phosphorylation *in cis*.

### C-terminal Domains of G1 Cyclins Confer F-box Protein Specificity

**Figure 5:**
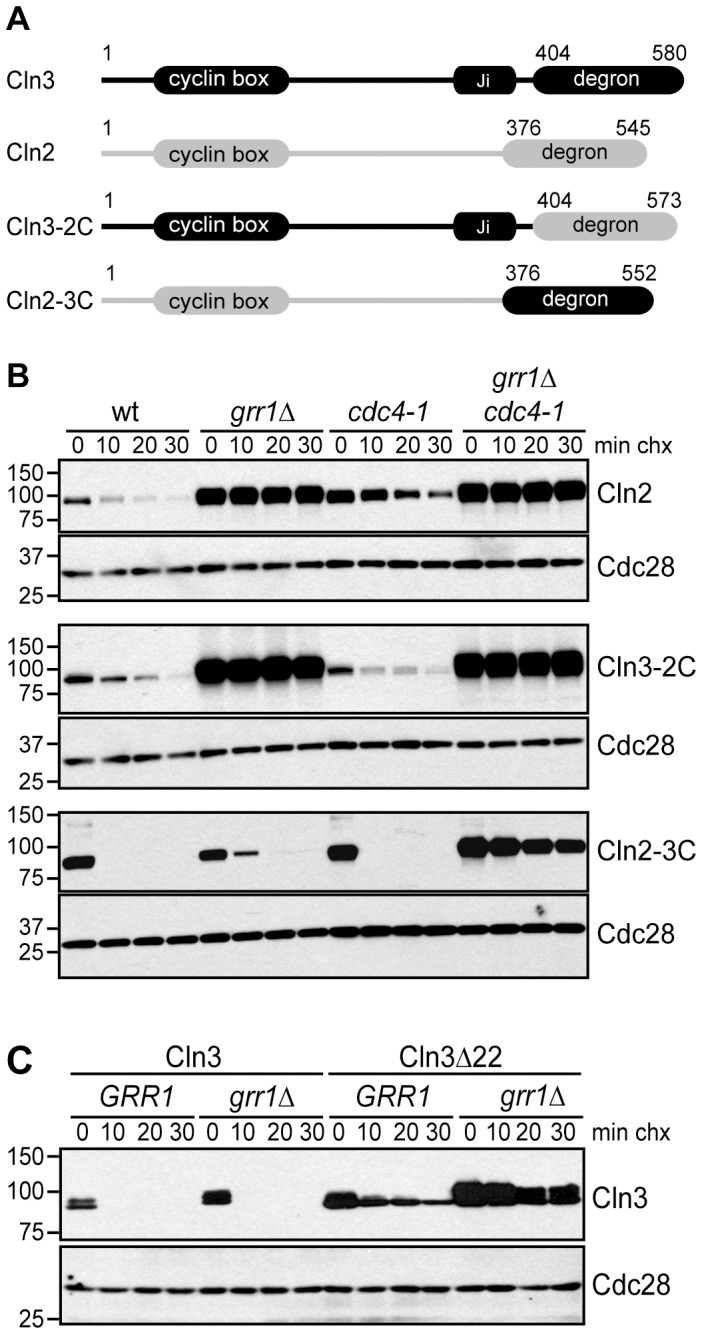
The C-terminal domains of G1 cyclins confer F-box protein specificity. (A) Diagram showing the domain organization of Cln3 and Cln2, as well as chimeric proteins. Cln3 sequence is shown in black, Cln2 sequence in grey. Domains labeled degron refer to C-terminal sequences that have been demonstrated to confer Grr1- or Grr1/Cdc4-dependent degradation, which are exchanged in the chimeric proteins. (B) Cycloheximide-chase assay of Cln2-13Myc, Cln3-2C-13Myc and Cln2-3C-13Myc in wild-type (wt), *grr1Δ*, *cdc4-1*, and *grr1Δ cdc4-1* cells, after shifting to the non-permissive temperature for 2 hours, followed by treatment with cycloheximide for the indicated number of minutes (min chx). Levels of Myc-tagged proteins and Cdc28 are shown. (C) Cycloheximide-chase assay of Cln3-13Myc and Cln3Δ22-13Myc in wild-type (*GRR1*) and *grr1Δ* cells. Levels of Myc-tagged Cln3 proteins and Cdc28 are shown after incubation with cycloheximide for the indicated number of minutes (min chx). Immunofluorescence images confirming the cytoplasmic localization of Cln3Δ22 are shown in Figure S3. For all gels, molecular weight markers are indicated at the left.

Although Cdc4 and Grr1 have both been shown to bind and ubiquitinate Cdk-phosphorylated proteins, they are thought to recognize non-overlapping sets of targets [36]. This raises the question of what is unique about Cln3 that enables it to be recognized by both F-box proteins. To address this issue, we compared Cln3 and the related cyclin Cln2, which is targeted for degradation exclusively by Grr1 [9]. Like Cln3, Cln2 has an N-terminal cyclin box and a C-terminus that includes a PEST domain and Cdk phosphorylation sites (Figure 5A; [37]). Moreover, the 169 C-terminal amino acids of Cln2 constitute a transferrable degron, which can direct Grr1-mediated degradation of a heterologous protein [38]. Together, this suggests that all of the FBP specificity resides within the C-terminal domains of both cyclins.

To test this possibility, chimeric proteins were created by exchanging the C-terminal degron domains of Cln2 and Cln3 (Figure 5A) and the stability of these chimeric proteins was assayed in strains lacking Grr1, Cdc4, or both FBPs. As expected for a Grr1 target, Cln2 was degraded rapidly in wild-type cells, but completely stable in *grr1Δ* cells (Figure 5B, top panels). Moreover, Cln2 was degraded in *cdc4-1* cells, although the protein level was higher overall (which was expected because *cdc4-1* cells arrest in G1/S phase when *CLN2* transcription peaks). In contrast to Cln2, Cln3 was not affected by loss of either Grr1 or Cdc4, but was completely stabilized in *grr1Δ cdc4-1* cells (Figure 2B). However, when the C-terminus of Cln3 was replaced with the C-terminus of Cln2 (Cln3-2C), the Cln3 degradation profile was nearly identical to that of Cln2 (Figure 5B, middle panels). In wild-type cells, the half-life of Cln3-2C was longer than Cln3 (compare Figure 2B to Figure 4B) and, importantly, Cln3-2C was completely stable in *grr1Δ* cells, demonstrating that it could no longer be targeted by Cdc4. When the C-terminus of Cln2 was replaced with the C-terminus of Cln3 (Cln2-3C), the opposite result was observed (Figure 5B, bottom panels). In wild-type cells, Cln2-3C was considerably less stable than Cln2. In addition, unlike Cln2, Cln2-3C was degraded in *grr1Δ* cells (although it was slightly more stable than in wild-type cells). However, Cln2-3C was stable in *grr1Δ cdc4-1* cells. Together, these data demonstrate the C-termini of Cln2 and Cln3 confer their FBP specificity, and indicate that there is a unique feature in the Cln3 C-terminus that promotes Cdc4-mediated turnover.

### Subcellular Localization Regulates G1 Cyclin Degradation

Our analysis of Cln3 truncation mutants demonstrated that the last 22 amino acids of Cln3 are important for interaction with Cdc4 but not Grr1 (Figure 3A). Interestingly, this 22 amino acid sequence in Cln3 includes a bipartite nuclear localization signal (NLS), which is important for Cln3 nuclear localization (Figure S3; [21], [22]). In contrast, Cln2 does not have an NLS motif and it is predominantly cytoplasmic [22], [28]. This raises the possibility that the localization of G1 cyclins may also contribute to FBP specificity *in vivo*. The localization of the FBPs supports this model: Grr1 is both cytoplasmic and nuclear, whereas Cdc4 is exclusively nuclear [39]. Therefore, since Cln2 is primarily cytoplasmic, it may only be accessible to Grr1. In contrast, since Cln3 is primarily nuclear, this may enable targeting by both Grr1 and Cdc4. Consistent with this model, the Cln3Δ22 mutant, which lacks the NLS sequence and is primarily cytoplasmic (Figure S3; [22]), is almost completely stable in *grr1Δ* cells (Figure 5C). Notably, we find that Cln3Δ22 is considerably more stable than full-length Cln3 expressed in cells lacking Cdc4 (compare Figure 5C and Figure 2B), despite that fact that it can bind to Grr1 as well as the full-length protein in whole cell extracts (Figure 3A). This suggests that nuclear Cln3 is more susceptible to degradation than cytoplasmic Cln3, perhaps because most cytoplasmic Cln3 is tethered to the ER and not fully active [23].

**Figure 6:**
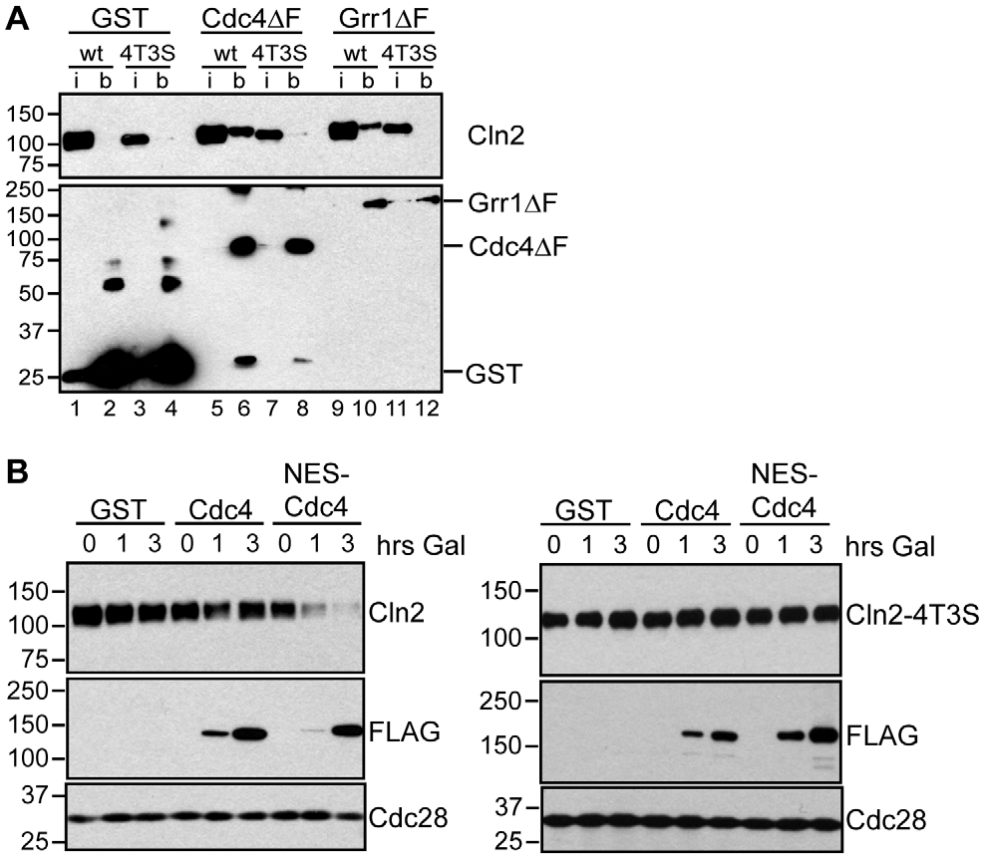
Cdc4 can target Cln2 for degradation upon co-localization. (A) Cdk-phosphorylated Cln2 interacts with Cdc4 and Grr1. Myc and GST Western blots showing pull-down of GST, GST-Cdc4ΔF and GST-Grr1ΔF proteins from *grr1Δ* cells expressing Cln2-13Myc (wt) or Cln2-4T3S-13Myc (4T3S). 2% input (i) and glutathione-sepharose bound proteins (b) are shown. (B) Expression of cytoplasmic Cdc4 downregulates Cdk-phosphorylated Cln2. Western blot showing levels of Cln2-13Myc, or Cln2-4T3S-13Myc, in *grr1Δ* cells after induction of GST, GST-Cdc4-FLAG, or GST-NES-Cdc4-FLAG expression following the addition of galactose for the indicated number of hours. Levels of FLAG-tagged proteins and Cdc28 are also shown. For all gels, molecular-weight markers are indicated at the left.

Another prediction from our data is that Cdc4 may be capable of targeting Cln2 for degradation, but does not do so *in vivo* only because Cln2 is predominantly localized to the cytoplasm. To test this idea, we first tested whether Cln2 could interact with Cdc4. Importantly, Cln2 associated with both Cdc4ΔF and Grr1ΔF proteins in extracts (Figure 6A, lanes 6 & 10). Moreover, Cln2-4T3S, a stable Cln2 protein that has mutations in 7 Cdk-consensus sites [37], was unable to bind to Grr1 or Cdc4 (Figure 6A, lanes 8 &12), suggesting that Cdc4 and Grr1 bind to similar Cdk-phosphorylated epitopes. In addition, we found that the third G1 cyclin, Cln1, also interacted with both Cdc4 and Grr1 (Figure S4). Thus, like Cln2, Cln1 is a potential target of both Cdc4 and Grr1, but may be regulated exclusively by Grr1 *in vivo* by virtue of its cytoplasmic localization.

Since Cdc4 interacts with Cln1 and Cln2 in extracts, this suggests that Cdc4 might be capable of targeting all three G1 cyclins *in vivo*, if it were localized in the same subcellular compartment as all three cyclins. To test this, we utilized a previously characterized Cdc4 protein (NES-Cdc4) that is fused to a nuclear export signal and localizes to the cytoplasm [39]. Expression of NES-Cdc4 in *grr1Δ* cells led to decreased Cln2 protein levels, whereas expression of wild-type Cdc4 had no effect (Figure 6B). In addition, this downregulation was dependent upon Cdk-phosphorylation of Cln2, since levels of Cln2-4T3S were unaffected by NES-Cdc4 expression (Figure 6B). Together, these data show that localization of yeast G1 cyclins determines which of the FBPs can target each *in vivo*, and that the binding specificities of the FBPs do not dictate which proteins are *in vivo* targets.

### Genetic Interaction between *GRR1* and *CDC4*

**Figure 7:**
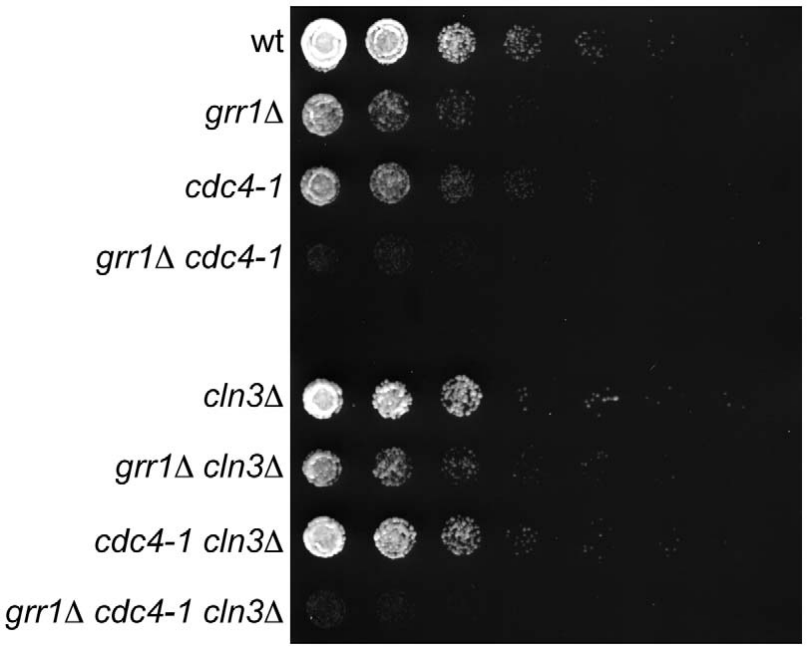
Genetic interaction between *CDC4* and *GRR1*. 5-fold dilutions of cells with the indicated genotypes were grown at 23°C (the permissive temperature for *cdc4-1*). All strains are *rgt1Δ*, which alleviates the glucose repression defect in *grr1Δ* strains. Note that *grr1Δ cdc4-1* cells are growing, but at a much slower rate than single mutant strains. Plates are shown at an early time point when wild-type and single mutant strains have not reached saturation, to better illustrate the differences in growth rates among the strains.

The finding that Cdc4 and Grr1 can bind to some of the same Cdk-phosphorylated proteins, and that they redundantly target Cln3 for degradation *in vivo*, suggests that these two FBPs may share a number of overlapping targets that are important for cell cycle progression. In support of this possibility, we found that cells carrying a *GRR1* deletion and having compromised Cdc4 function (*grr1Δ cdc4-1*, grown at the permissive temperature for *cdc4-1*) demonstrate a synergistic growth defect well beyond that predicted for a combination of the individual (minor) growth phenotypes (Figure 7). This type of negative genetic interaction is consistent with Cdc4 and Grr1 having partially redundant roles in one or more common cellular functions [40]. Importantly, deletion of *CLN3* did not reverse this growth defect (Figure 7), supporting the idea that additional proteins are redundantly targeted by Cdc4 and Grr1. Alternatively, it is possible that Cdc4 and Grr1 have specific substrates that cause a synergistic growth defect when their levels are elevated. However, the large number of unstable, Cdk-phosphorylated proteins in the cell [13], [14], [41], combined with the ability of Grr1 and Cdc4 to bind some targets in common, raises the intriguing possibility that these two FBPs target a significant fraction of the cell cycle proteome for degradation.

## Discussion

Cln3 turnover is essential for accurate entry into the cell cycle and for proper cell size control [24]–[26], however the identity of the ubiquitin ligase that targets Cln3 for destruction has remained a long-standing question. Here, we show that Cln3 is redundantly targeted by two F-box proteins with key cell cycle-regulatory roles, Cdc4 and Grr1. Interestingly, inactivation of either FBP alone has no detectable effect on Cln3 expression or half-life, yet Cln3 is completely stable in double mutant cells (Figure 2B–2C). This result is quite surprising because, although redundant regulation of degradation has been previously found for other proteasome substrates in both yeast and mammals [42]–[44], in most cases elimination of one ubiquitin ligase or the other leads to a partial stabilization of the substrate, which we do not observe with Cln3. This may be because targeting of these other substrates by two ligases is only partially redundant in the sense that the ligases may recognize different epitopes on the substrate, or respond to different physiological cues.

In the case of Cln3, our data suggests that both Cdc4 and Grr1 recognize the Cdk-phosphorylated C-terminus of Cln3 (Figure 4). However, the two FBPs do not recognize identical epitopes. Cdc4 targeting requires three phosphosites at the extreme C-terminus of Cln3, whereas mutation of these sites have no effect on targeting by Grr1 (Cln3-3A, Figure 4D, bottom panels). Interestingly, mutation of a second cluster of five phosphosites partially interferes with targeting by both Cdc4 and Grr1 (Cln3-5A, Figure 4D, top panels). This suggests that the two FBPs may also interact with some overlapping residues. Alternatively, these five phosphosites may be required as priming sites that promote the phosphorylation of the three C-terminal sites that Cdc4 requires, in a mechanism similar to what has recently been demonstrated for the Cdc4-specific target Sic1 [35]. Further work will be required to dissect the mechanism of targeting by each FBP. However, since the Cln3 C-terminus is likely to be constitutively phosphorylated by Cdk *in cis* (Figure 4E), and we have not been able to identify any condition when only one FBP targets Cln3, this suggests that Cdc4 and Grr1 are truly redundant for Cln3 degradation.

The fact that there is no significant change in Cln3 levels in either single mutant, along with the observed genetic interaction between *CDC4* and *GRR1* (Figure 7), raises the possibility that there are additional redundant targets of Cdc4 and Grr1 that have not yet been identified. A likely possibility is that other Cdk-phosphorylated, nuclear proteins are dual-regulated targets. Given the large number of Cdk-phosphorylated proteins that are expressed in a cell cycle-dependent pattern [12], [13], [19], [20], it is possible that Cdc4 and Grr1 recognize a much larger fraction of all cell cycle-regulated proteolysis than was previously recognized. However, Cdk-phosphorylation cannot be the only factor that determines whether a protein can be an *in vivo* substrate of both Grr1 and Cdc4, since established Cdk-phosphorylated Cdc4 targets, such as Sic1 and Far1, cannot be targeted by Grr1 *in vivo* or *in vitro* [4], [39]. In addition, we find that deletion of *SIC1* does not reverse the synthetic sickness of *grr1Δ cdc4-1* strains (data not shown). Whether the synthetic growth defect observed for *cdc4* and *grr1* is due to upregulation of a single common target protein that is crucial for proliferation, or multiple targets (either shared or specific) that each play a modest role in the cell cycle is a key question requiring further study.

In contrast to Cln3, which is targeted by both Cdc4 and Grr1, the related cyclins Cln1 and Cln2 are targeted exclusively by Grr1 *in vivo* [9]. Interestingly, we find that Cdc4 can interact with both Cln1 and Cln2 in extracts (Figure S4; Figure 6). Moreover, previous studies with recombinant proteins reported a weak association between Cln2 and Cdc4 [4] and demonstrated that Cdc4 can ubiquitinate Cln2 *in vitro* [39]. Interestingly, since Cln2 has been shown to phosphorylate Whi5 in the nucleus [45], this suggests that Cdc4 may have an unappreciated role in targeting a small but important nuclear fraction of Cln1/2. Alternatively, the small fraction of nuclear Cln2 may be protected from degradation in some way. In addition, these data indicate that FBPs do not necessarily have the exquisite binding specificity for substrates that has been proposed, and that co-localization of FBPs and substrates also contributes to substrate specificity *in vivo*. In the future it will be of interest to investigate whether any other Grr1 substrates can interact with Cdc4, or be targeted by Cdc4 upon co-localization.

Importantly, the finding that FBP specificity for G1 cyclins depends upon the localization of cyclins may aid in our understanding of the mechanism of degradation of the mammalian cyclin D1 protein. Several FBPs have been implicated in cyclin D1 degradation [46]–[51], and it is possible that it is regulated by similar redundant mechanisms. It is interesting to note that degradation of the furthest upstream G1 cyclins appears to be quite complex and include redundancy. Since both Cln3 and cyclin D1 are crucial regulators that act as sensors of extracellular growth signals and trigger entry into the cell cycle, cells may have evolved redundant mechanisms to degrade these cyclins in order to buffer cells against dramatic fluctuations in cyclin synthesis and inappropriate cell cycle entry.

## Materials and Methods

### Yeast Strains and Plasmids

A complete list of strains, including the specific experiments each was used in, is provided in Table S1. Unless otherwise indicated, all strains are in the S288c background and have a 13Myc-HIS3MX C-terminal tagging cassette integrated at the *CLN3* or *CLN2* genomic locus. Strains carrying point mutations in phosphorylation sites were generated by integrating a PCR product containing the desired mutations, the 13Myc tag, and the HIS3MX marker at the *CLN3* or *CLN2* genomic locus. The integration of each mutation was then confirmed by sequencing.

Plasmids expressing GST and GST-tagged Grr1 have been described previously [32]. To construct pYES2-GST-CDC4, the *CDC4* sequence was amplified from genomic DNA by PCR and cloned into the pYES2-GST vector. pYES2-CDC4ΔF was constructed similarly, except that the sequence corresponding to amino acids 323–475 was amplified and cloned into pYES2-GST. To construct the GST-NES-CDC4 plasmid, a DNA fragment including the NES and the *CDC4* N-terminal sequences was subcloned from pBM138 (provided by Matthias Peter, [39]) into pYES2-GST-CDC4.

All strains were grown in YM-1 complete medium with 2% dextrose, with the exception of strains carrying GST plasmids, which were grown in C medium lacking uracil with 2% dextrose or raffinose [15]. To arrest cells in G1, 20 µg/ml alpha-factor (United Biochemical Research, Inc.) was added for 2–3 hours. To arrest cells in mitosis, 10 µg/ml nocodazole (US Biological) was added to cells for 2 hours. Strains carrying temperature-sensitive alleles of *CDC53* or *CDC4* were grown at 23°C and shifted to 37°C for 2 hours to inactivate the respective proteins. Strains carrying tetracycline-regulated alleles of *CDC53*, *CDC4* and *CDC34* were treated with 10 µg/ml doxycycline (EMD Biosciences) for 8 hours to shut off transcription from the tetracycline-regulated promoters (Figure S1D). For GST-pulldown assays to analyze Cln3 binding (Figure 3A; Figure 4C), *grr1Δ cdc4-1* strains carrying GST plasmids were grown in 2% raffinose and then induced by the addition of 2% galactose for 20–22 hours. For GST-pulldown assays to analyze Cln1 and Cln2 binding (Figure 6A; Figure S4), *grr1Δ* strains carrying GST plasmids were grown in 2% raffinose and then induced by the addition of 2% galactose for 8 hours. To examine levels of Cln2 and Cln2-4T3S following expression of GST, GST-CDC4, and GST-NES-CDC4 (Figure 6B), *grr1Δ* cells carrying GST plasmids were grown in 2% raffinose and expression was induced by the addition of 0.05% galactose for the indicated number of hours.

### Western Blotting

Equivalent optical densities of cells were pelleted, lysed in pre-heated SDS sample buffer (50 mM Tris pH 7.5, 5 mM EDTA, 5% SDS, 10% glycerol, 0.5% β-mercaptoethanol, bromophenol blue, 1 µg/ml leupeptin, 1 µg/ml bestatin, 1 mM benzamidine, 1 µg/ml pepstatin A, 17 µg/ml PMSF, 5 mM sodium fluoride, 80 mM β-glycerophosphate and 1 mM sodium orthovanadate) and heated to 95°C for 5 minutes. Glass beads were then added and samples were bead-beat for 3 minutes in a Mini-BeadBeater-96 (Biospec), followed by centrifugation. For cycloheximide assays with two minute time points, cell pellets were lysed in cold TCA buffer (10 mM Tris pH 8.0, 10% trichloroacetic acid, 25 mM ammonium acetate, 1 mM EDTA) and incubated on ice. Samples were then centrifuged and the pellets resuspended in Resuspension Solution (0.1 M Tris pH 11.0, 3% SDS). Samples were heated to 95°C for 5 minutes, allowed to cool to room temperature, and clarified by centrifugation. Supernatants were added to 4× SDS-PAGE Sample Buffer (0.25 M Tris pH 6.8, 8% SDS, 40% glycerol, 20% β-mercaptoethanol) and heated to 95°C for 5 minutes. Extracts were then subjected to SDS–polyacrylamide gel electrophoresis (SDS–PAGE), followed by transfer to nitrocellulose membranes, and Western blotting with antibodies against Myc (Clone 9E10, Covance) Cdc28 (sc-6709, Santa Cruz Biotechnology), Clb2 (sc-9071, Santa Cruz Biotechnology), Cdc53 (sc-6717, Santa Cruz Biotechnology), Flag (Clone M2, Sigma) and GST (Clone 4C10, Covance).

### Cycloheximide-Chase Assays

Cells were grown to mid-log phase, or arrested as indicated, then treated with 50 µg/ml cycloheximide. Samples were fixed for flow cytometry, and cell pellets from equivalent optical densities of cells were collected for Western blotting at indicated time points.

### Cell Cycle Analysis

Cells were fixed with 70% ethanol and stored at 4°C overnight. Cells were then sonicated, treated with 0.25 mg/ml RNase A for 1 hour at 50°C, followed by digestion with 0.125 mg/ml Proteinase K for 1 hour at 50°C and labeling with 1 µM Sytox Green (Invitrogen). Data was collected using a FACScan (Becton Dickinson) and analyzed with FlowJo (Tree Star, Inc.) software.

### GST-Pulldown Assays

Cell pellets containing 20–30 optical densities of cells were lysed in HEPES lysis buffer (25 mM HEPES pH 7.6, 400 mM NaCl, 0.2% Triton X-100, 1 mM EDTA, 10% glycerol, 1 µg/ml leupeptin, 1 µg/ml bestatin, 1 mM benzamidine, 1 µg/ml pepstatin A, 17 µg/ml PMSF, 5 mM sodium fluoride, 80 mM β-glycerophosphate and 1 mM sodium orthovanadate) by bead beating in a cold block for 3 minutes and clarified by centrifugation at 4°C. Extracts were incubated with 20 µl of a 50% slurry of glutathione-sepharose 4B in lysis buffer (GE Healthcare), while rotating at 4°C for 2 hours. Beads were collected by centrifugation and washed seven times with 1 ml lysis buffer. Proteins were eluted by boiling in 2× SDS-PAGE sample buffer and analyzed by Western blotting against the GST and Myc tags. 2% input of each extract is also shown.

### Immunofluorescence

Cells expressing Myc-tagged Cln3 proteins were fixed in 3.7% formaldehyde for 1 hour at 23°C followed by two washes in potassium phosphate buffer (83 mM K_2_HPO_4_, 17 mM KH_2_PO_4_, pH 7.5) and one wash in sorbitol phosphate buffer (1.2 M sorbitol, 83 mM K_2_HPO_4_, 17 mM KH_2_PO_4_, pH 7.5). Cells were then spheroplasted with zymolyase and adhered to poly-L-lysine coated slides. Cells on slides were permeabilized with methanol and acetone, then blocked in PBS-BSA (10 mg/ml bovine serum albumin, 0.04 M K_2_HPO_4_, 0.01 M KH_2_PO_4_, 0.15 M NaCl, 0.1%NaN_3_). Cells were then incubated with rabbit anti-Myc antibody (sc-789, Santa Cruz Biotechnology) overnight, followed by 5 washes in PBS-BSA, and incubation with Alexafluor 488-conjugated goat anti-mouse secondary antibody (Invitrogen). Cells were again washed 5 times with PBS-BSA, then stained with 1 µg/ml DAPI and mounted with ProLong Gold Antifade reagent (Invitrogen). Microscopy was carried out using a Zeiss Axioskop 2 fluorescence microscope with an 100× 1.3NA Plan-NEOFLAUR objective. Images were taken with a RT Monochrome SPOT camera (Diagnostic Instruments, Inc.) and accompanying software. Image analysis was done with Adobe Photoshop software. All images were captured for the same exposure times and adjustments to contrast and brightness were performed equally on all panels.

## Supporting Information

Figure S1 Cell cycle regulation of Cln3. (A) Quantification of Cln3 levels from Figure 1A by densitometry. The ratios of Cln3 signal to Cdc28 signal for each time point are plotted on a log2 scale. (B) Cell cycle profile of cells arrested in G1 with alpha-factor, or in mitosis with nocodazole, prior to the addition of cycloheximide (control for Figure 1B). (C) Cell cycle profiles of wild-type (*CDC53*) and *cdc53-1* cells after shifting to the restrictive temperature for 2 hours, prior to the addition of cycloheximide (control for Figure 1C). (D) Wild-type (wt) and strains expressing tetracycline-regulated *CDC53*, *CDC34* and *CDC4* genes were treated with doxycycline for 8 hours to shut off transcription, and then cycloheximide was added for the indicated number of minutes (min chx). Levels of Cln3-13Myc, Cdc53 and Cdc28 are shown. For all gels, molecular-weight markers are indicated at the left. Cell cycle profiles of doxycycline-treated cells, prior to the addition of cycloheximide, are shown on the right.
(TIF)

Figure S2 Regulation of Cln3 degradation. (A) Western blot of Cln3-13Myc in *grr1Δ cdc4-1* cells expressing GST (empty) or F-box proteins tagged with an N-terminal GST and a C-terminal FLAG tag (Cdc4, Cdc4ΔF, Grr1, Grr1ΔF). All proteins are expressed from the *GAL1* promoter. Levels of Cln3, FLAG-tagged fusion proteins and Cdc28 are shown. (B) Diagram of the Cln3 C-terminus, corresponding to amino acids 404–580. PEST domains are shown in blue boxes and numbered 1–5. Minimal Cdk consensus sites (S/TP), T420, S447, T455, S462, S464, T478, S514, T517, and T520, are shown as yellow stars, the single full Cdk consensus site (S/TPxK), S468, is shown as a red star. The nuclear localization signal (NLS) is at the extreme C-terminus and is indicated by a purple box. Positions of truncation mutants (from Figure 3) are shown with dashed lines. Groups of consensus sites mutated to alanine in Figure 4 are indicated above the diagram. (C) Mutation of the full Cdk-consensus site, S468, has a small effect on Cln3 stability. Cycloheximide-chase assay showing levels of Cln3-13Myc and Cln3-S468A-13Myc after the addition of cycloheximide for the indicated number of minutes (min chx). Cdc28 is shown as a loading control. For all gels, molecular-weight markers are indicated at the left. (D) The Cln3-S468A mutation has no detectable effect on the cell cycle. Cell cycle position of cells from (C) before the addition of cycloheximide. (E) Cell cycle position of cells from Figure 3E. Only the Cln3-1 truncation has a detectable effect on cycle position. (F) Cell cycle position of wild-type or *grr1Δ* cells expressing Cln3 mutant proteins.
(TIF)

Figure S3 Localization of Cln3 proteins. Immunofluorescence of 13Myc-tagged Cln3 proteins and corresponding DAPI images. Note that Cln3 is primarily nuclear in wild-type (*GRR1 CDC4*) and *grr1Δ cdc4-1* cells, whereas Cln3Δ22 is primarily cytoplasmic in wild-type and *grr1Δ* cells. Also, the stable Cln3-9A mutant is primarily nuclear. Scale bar represents 5 µm.
(TIF)

Figure S4 Cln1 interacts with both Cdc4 and Grr1. (A) Cln1 is targeted by Grr1 *in vivo*. Cycloheximide chase assay showing levels of Cln1-13Myc after the addition of cycloheximide for the indicated number of minutes (min chx). (B) Cln1 interacts with both Grr1 and Cdc4 in extracts. Myc and GST Western blots showing pull-downs of GST, GST-Cdc4ΔF and GST-Grr1ΔF proteins from *grr1Δ* cells expressing Cln1-13Myc. 2% input and glutathione-sepharose bound proteins (bound) are shown. For all gels, molecular-weight markers are indicated at the left.
(TIF)

Table S1 Strains list.
(PDF)
