# Supplementary figures and images for: F-Box Protein Specificity for G1 Cyclins Is Dictated by Subcellular Localization
Source: PLoS Genet. 2012 Jul 26;8(7):e1002851. doi: 10.1371/journal.pgen.1002851 (PMC3405998; doi:10.1371/journal.pgen.1002851)

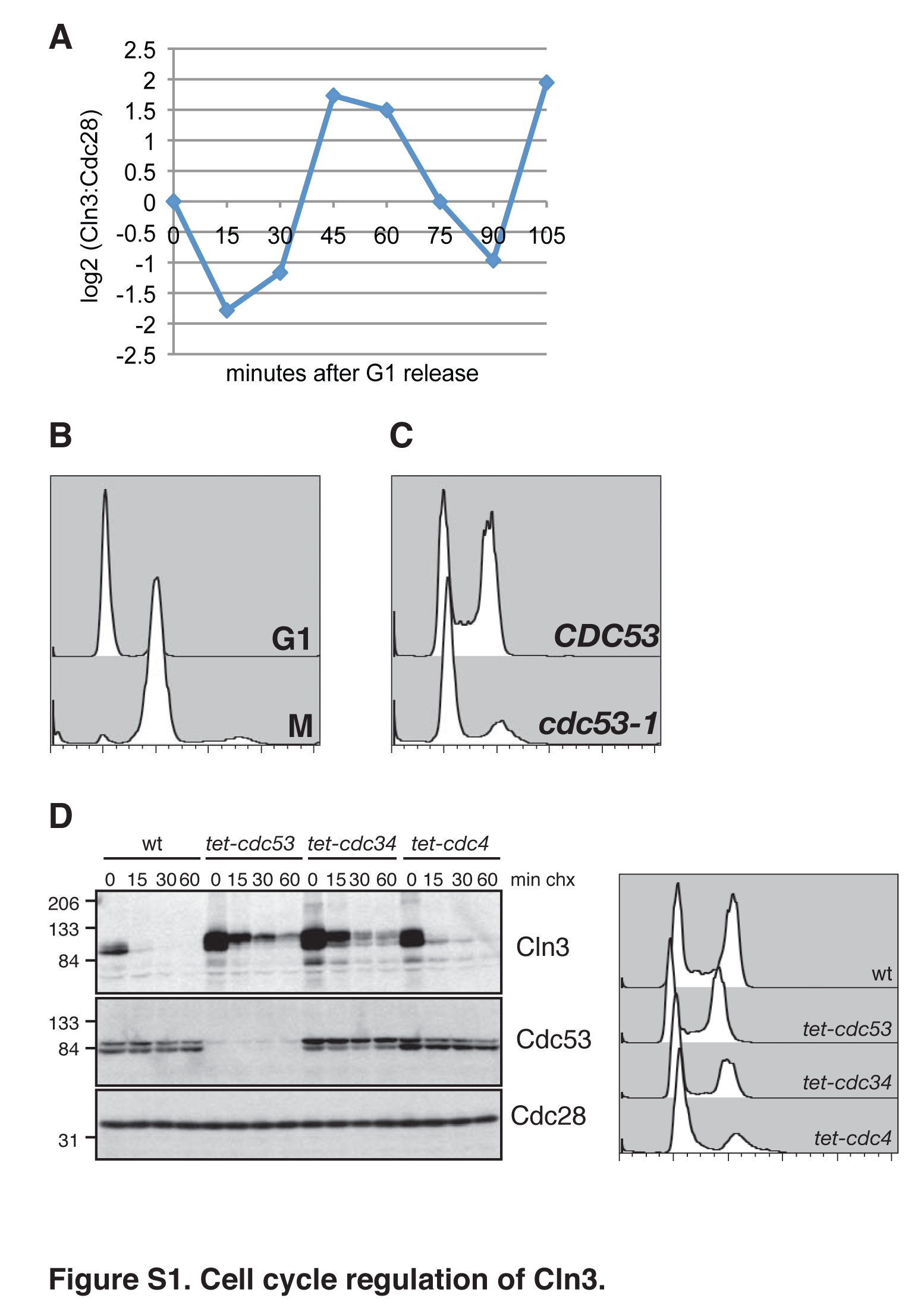

Supplement: Figure S1 — Cell cycle regulation of Cln3. (A) Quantification of Cln3 levels from Figure 1A by densitometry. The ratios of Cln3 signal to Cdc28 signal for each time point are plotted on a log2 scale. (B) Cell cycle profile of cells arrested in G1 with alpha-factor, or in mitosis with nocodazole, prior to the addition of cycloheximide (control for Figure 1B). (C) Cell cycle profiles of wild-type (CDC53) and cdc53-1 cells after shifting to the restrictive temperature for 2 hours, prior to the addition of cycloheximide (control for Figure 1C). (D) Wild-type (wt) and strains expressing tetracycline-regulated CDC53, CDC34 and CDC4 genes were treated with doxycycline for 8 hours to shut off transcription, and then cycloheximide was added for the indicated number of minutes (min chx). Levels of Cln3-13Myc, Cdc53 and Cdc28 are shown. For all gels, molecular-weight markers are indicated at the left. Cell cycle profiles of doxycycline-treated cells, prior to the addition of cycloheximide, are shown on the right. (TIF) [file pgen.1002851.s001.tif]

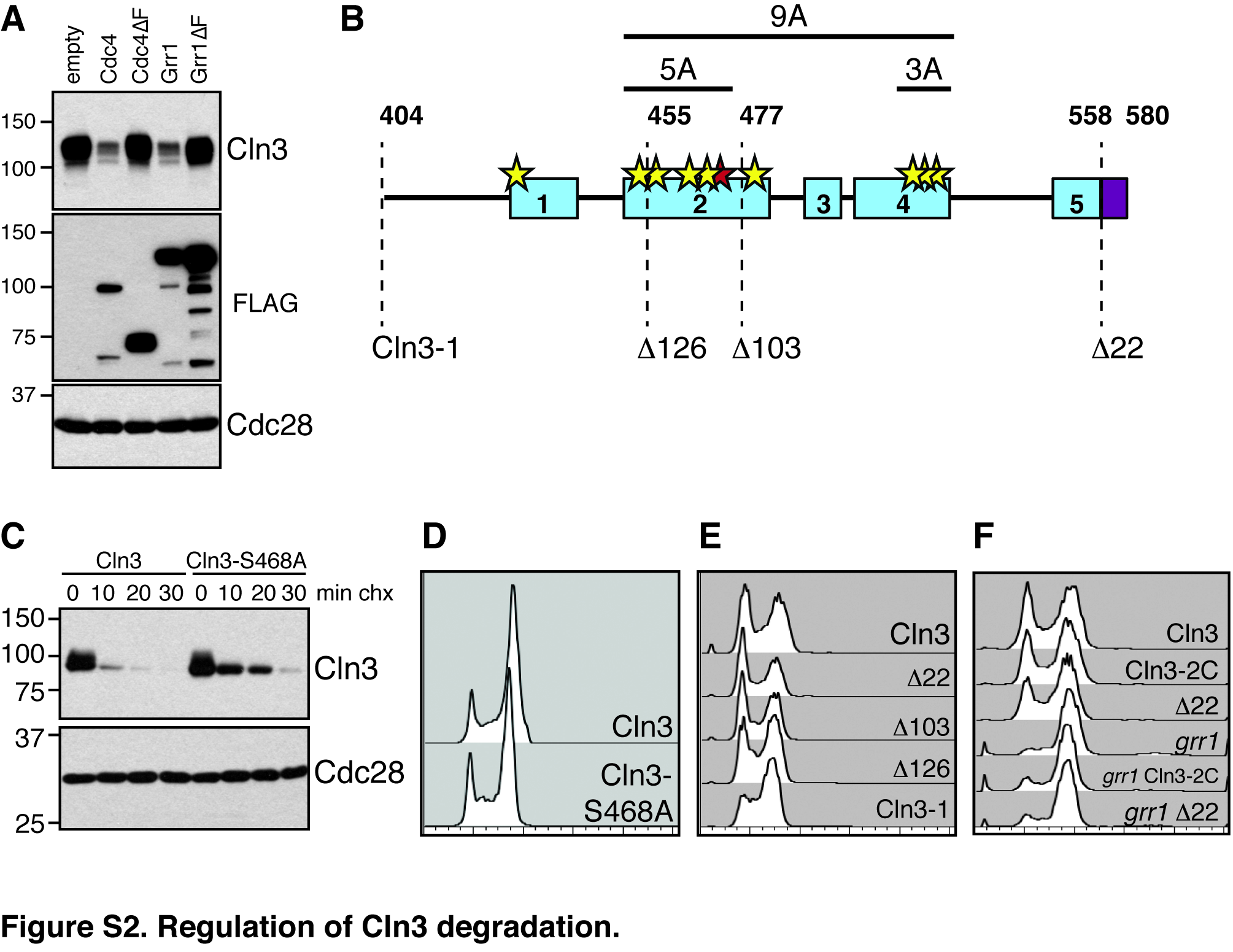

Supplement: Figure S2 — Regulation of Cln3 degradation. (A) Western blot of Cln3-13Myc in grr1Δ cdc4-1 cells expressing GST (empty) or F-box proteins tagged with an N-terminal GST and a C-terminal FLAG tag (Cdc4, Cdc4ΔF, Grr1, Grr1ΔF). All proteins are expressed from the GAL1 promoter. Levels of Cln3, FLAG-tagged fusion proteins and Cdc28 are shown. (B) Diagram of the Cln3 C-terminus, corresponding to amino acids 404–580. PEST domains are shown in blue boxes and numbered 1–5. Minimal Cdk consensus sites (S/TP), T420, S447, T455, S462, S464, T478, S514, T517, and T520, are shown as yellow stars, the single full Cdk consensus site (S/TPxK), S468, is shown as a red star. The nuclear localization signal (NLS) is at the extreme C-terminus and is indicated by a purple box. Positions of truncation mutants (from Figure 3) are shown with dashed lines. Groups of consensus sites mutated to alanine in Figure 4 are indicated above the diagram. (C) Mutation of the full Cdk-consensus site, S468, has a small effect on Cln3 stability. Cycloheximide-chase assay showing levels of Cln3-13Myc and Cln3-S468A-13Myc after the addition of cycloheximide for the indicated number of minutes (min chx). Cdc28 is shown as a loading control. For all gels, molecular-weight markers are indicated at the left. (D) The Cln3-S468A mutation has no detectable effect on the cell cycle. Cell cycle position of cells from (C) before the addition of cycloheximide. (E) Cell cycle position of cells from Figure 3E. Only the Cln3-1 truncation has a detectable effect on cycle position. (F) Cell cycle position of wild-type or grr1Δ cells expressing Cln3 mutant proteins. (TIF) [file pgen.1002851.s002.tif]

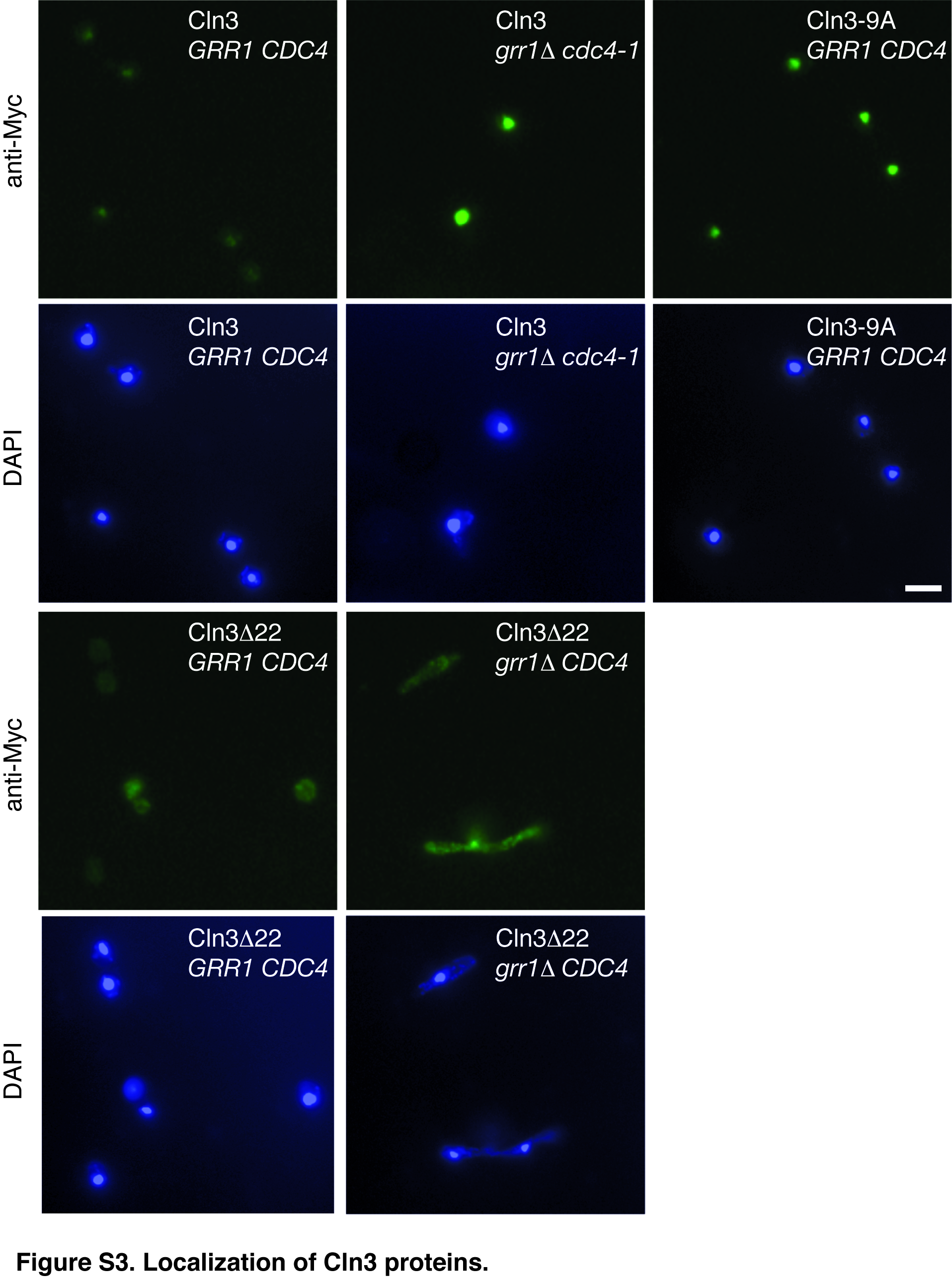

Supplement: Figure S3 — Localization of Cln3 proteins. Immunofluorescence of 13Myc-tagged Cln3 proteins and corresponding DAPI images. Note that Cln3 is primarily nuclear in wild-type (GRR1 CDC4) and grr1Δ cdc4-1 cells, whereas Cln3Δ22 is primarily cytoplasmic in wild-type and grr1Δ cells. Also, the stable Cln3-9A mutant is primarily nuclear. Scale bar represents 5 µm. (TIF) [file pgen.1002851.s003.tif]

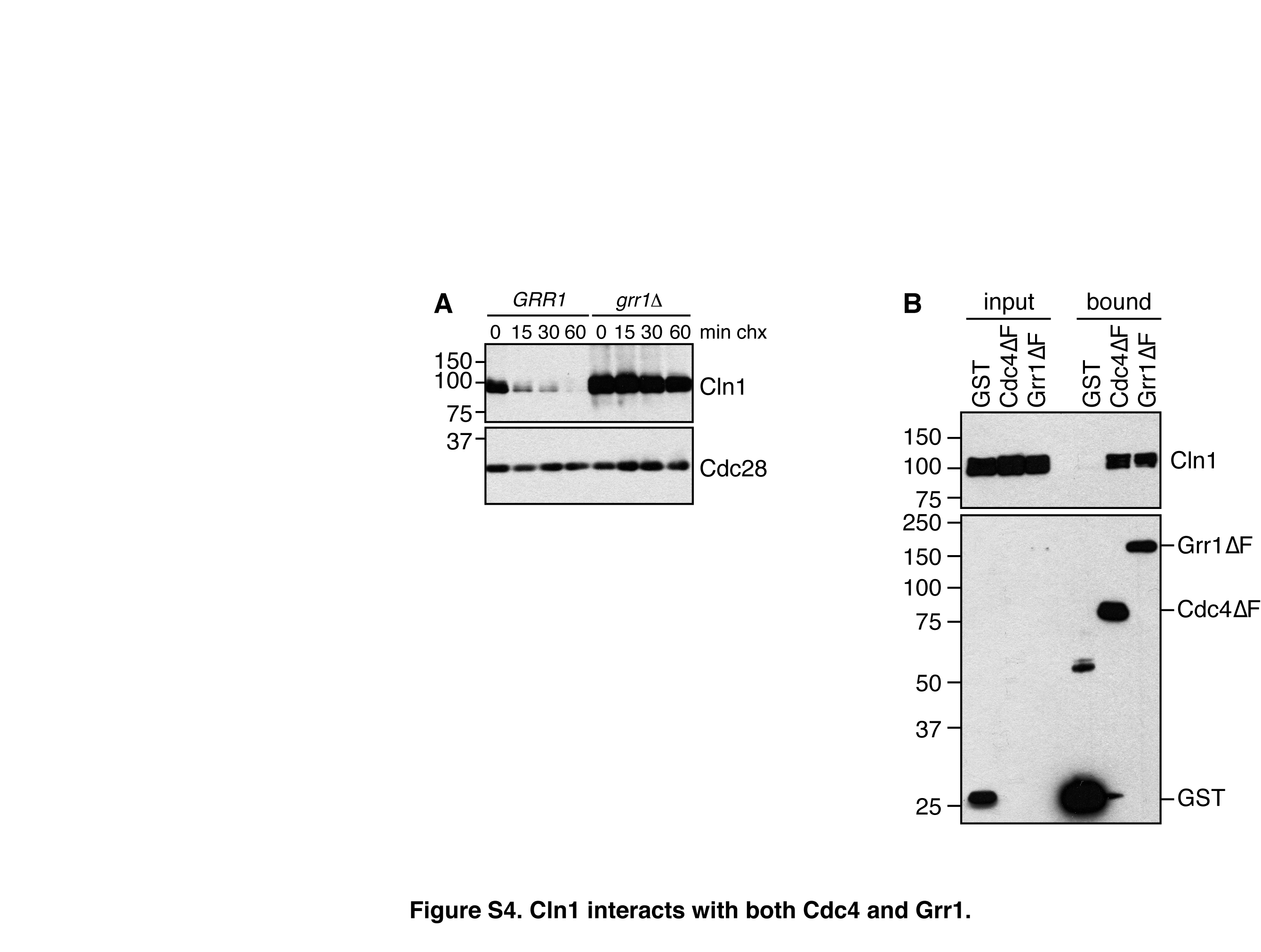

Supplement: Figure S4 — Cln1 interacts with both Cdc4 and Grr1. (A) Cln1 is targeted by Grr1 in vivo. Cycloheximide chase assay showing levels of Cln1-13Myc after the addition of cycloheximide for the indicated number of minutes (min chx). (B) Cln1 interacts with both Grr1 and Cdc4 in extracts. Myc and GST Western blots showing pull-downs of GST, GST-Cdc4ΔF and GST-Grr1ΔF proteins from grr1Δ cells expressing Cln1-13Myc. 2% input and glutathione-sepharose bound proteins (bound) are shown. For all gels, molecular-weight markers are indicated at the left. (TIF) [file pgen.1002851.s004.tif]
