# Supplementary material for: F-Box Protein Specificity for G1 Cyclins Is Dictated by Subcellular Localization
Source: PLoS Genet. 2012 Jul 26;8(7):e1002851. doi: 10.1371/journal.pgen.1002851 (PMC3405998; doi:10.1371/journal.pgen.1002851)
Supplement: Table S1 — Strains list. (PDF) [file pgen.1002851.s005.pdf]

Table S1. Strains List

Strain	Genotype	Figures
YJB240-1	*MATa his3Δ1 ura3Δ0 leu2Δ0 met15Δ0 CLN3-13MYC-HIS3*	1A-C, 2A
YJB362-1	*MATa his3Δ1 ura3Δ0 leu2Δ0 met15Δ0 CLN3-13MYC-HIS3 cdc53-1*	1C
YJB364-1	*MATa his3Δ1 leu2Δ0 met15Δ0 URA3::CMV-tTA CLN3-13MYC-HIS3*	S1D
YJB365-1	*MATa his3Δ1 leu2Δ0 met15Δ0 URA3::CMV-tTA cdc53p::kanR-tetO7-TATA CLN3-13MYC-HIS3*	S1D
YJB366-1	*MATa his3Δ1 leu2Δ0 met15Δ0 URA3::CMV-tTA cdc34p::kanR-tetO7-TATA CLN3-13MYC-HIS3*	S1D
YJB367-1	*MATa his3Δ1 leu2Δ0 met15Δ0 URA3::CMV-tTA cdc4p::kanR-tetO7-TATA CLN3-13MYC-HIS3*	S1D
YJB261	*MATa his3Δ1 ura3Δ0 leu2Δ0 lyp1Δ can1Δ: :STE2p-LEU2 CLN3-13MYC-HIS3 cos111Δ::ADHp-NLS-mRFP-URA3*	2A
YJB262	*MATa his3Δ1 ura3Δ0 leu2Δ0 lyp1Δ can1Δ: :STE2p-LEU2 CLN3-13MYC-HIS3 saf1Δ::ADHp-NLS-mRFP-URA3*	2A
YJB263	*MATa his3Δ1 ura3Δ0 leu2Δ0 lyp1Δ can1Δ: :STE2p-LEU2 CLN3-13MYC-HIS3 ydr131cΔ::ADHp-NLS-mRFP-URA3*	2A
YJB264	*MATa his3Δ1 ura3Δ0 leu2Δ0 lyp1Δ can1Δ: :STE2p-LEU2 CLN3-13MYC-HIS3 mfb1Δ::ADHp-NLS-mRFP-URA3*	2A
YJB265	*MATa his3Δ1 ura3Δ0 leu2Δ0 lyp1Δ can1Δ: :STE2p-LEU2 CLN3-13MYC-HIS3 ydr306c ::ADHp-NLS-mRFP-URA3*	2A
YJB266	*MATa his3Δ1 ura3Δ0 leu2Δ0 lyp1Δ can1Δ: :STE2p-LEU2 CLN3-13MYC-HIS3 das1Δ::ADHp-NLS-mRFP-URA3*	2A
YJB269	*MATa his3Δ1 ura3Δ0 leu2Δ0 lyp1Δ can1Δ: :STE2p-LEU2 CLN3-13MYC-HIS3 hrt3Δ::ADHp-NLS-mRFP-URA3*	2A
YJB270	*MATa his3Δ1 ura3Δ0 leu2Δ0 lyp1Δ can1Δ: :STE2p-LEU2 CLN3-13MYC-HIS3 mdm30Δ::ADHp-NLS-mRFP-URA3*	2A
YJB271	*MATa his3Δ1 ura3Δ0 leu2Δ0 lyp1Δ can1Δ: :STE2p-LEU2 CLN3-13MYC-HIS3 ufo1Δ::ADHp-NLS-mRFP-URA3*	2A
YJB272	*MATa his3Δ1 ura3Δ0 leu2Δ0 lyp1Δ can1Δ: :STE2p-LEU2 CLN3-13MYC-HIS3 dia2Δ::ADHp-NLS-mRFP-URA3*	2A
YJB273	*MATa his3Δ1 ura3Δ0 leu2Δ0 lyp1Δ can1Δ: :STE2p-LEU2 CLN3-13MYC-HIS3 ynl311cΔ::ADHp-NLS-mRFP-URA3*	2A
YJB274	*MATa his3Δ1 ura3Δ0 leu2Δ0 lyp1Δ can1Δ: :STE2p-LEU2 CLN3-13MYC-HIS3 ylr224wΔ::ADHp-NLS-mRFP-URA3*	2A
YJB275	*MATa his3Δ1 ura3Δ0 leu2Δ0 lyp1Δ can1Δ: :STE2p-LEU2 CLN3-13MYC-HIS3 ylr352wΔ::ADHp-NLS-mRFP-URA3*	2A
YJB278	*MATa his3Δ1 ura3Δ0 leu2Δ0 lyp1Δ can1Δ: :STE2p-LEU2*	2A

	CLN3-13MYC-HIS3 met30Δ::ADHp-NLS-mRFP-URA3 met4Δ::NatMX	
YJD31-1	*MATa his3Δ1 ura3Δ0 leu2Δ0 CLN3-13MYC-HIS3*	2B, 2C, 3A, 5C, S3
YJB426-2	*MATa his3Δ1 ura3Δ0 leu2Δ0 CLN3-13MYC-HIS3 grr1Δ::NLS-RFP-LEU2*	2B, 2C, 5C
YJD35-1	*MATa his3Δ1 ura3Δ0 leu2Δ0 CLN3-13MYC-HIS3 cdc4-1-Hyg*	2B, 2C
YJD43-1	*MATa his3Δ1 ura3Δ0 leu2Δ0 CLN3-13MYC-HIS3 grr1Δ::NLS-RFP-LEU2 cdc4-1-Hyg*	2B, S3
YBL156	*MATa ura3Δ0 leu2Δ0 his3Δ0 met15Δ0 +pYES2-GST-URA3*	2D
YBL157	*MATa ura3Δ0 leu2Δ0 his3Δ0 met15Δ0 +pGAL-CLN3-URA3*	2D
YBL158	*MATa ura3Δ0 leu2Δ0 his3Δ0 met15Δ0 grr1Δ::NLS-mRFP-LEU2 +pYES2-GST-URA3*	2D
YBL159	*MATa ura3Δ0 leu2Δ0 his3Δ0 met15Δ0 grr1Δ::NLS-mRFP-LEU2 +pGAL-CLN3-URA3*	2D
YBL160	*MATα ura3Δ0 leu2Δ0 his3Δ0 lys2Δ0 cdc4-1-Hyg +pYES2-GST-URA3*	2D
YBL161	*MATα ura3Δ0 leu2Δ0 his3Δ0 lys2Δ0 cdc4-1-Hyg +pGAL-CLN3-URA3*	2D
YJD32-1	*MATa his3Δ1 ura3Δ0 leu2Δ0 CLN3-13MYC-HIS3 rgt1Δ::KanMX*	S2C-D
YJD73-1	*MATa his3Δ1 ura3Δ0 leu2Δ0 CLN3-S468A-13MYC-HIS3 rgt1Δ::KanMX*	S2C-D
YJB434-1	*MATa ura3Δ0 leu2Δ0 his3Δ0 met15Δ0 CLN3Δ22-13MYC-HIS3*	3A, 5C, S3
YJB503	*MATa ura3Δ0 leu2Δ0 his3Δ0 met15Δ0 Cln3-1-13Myc-His3MX*	3A
YJB505	*MATa ura3Δ0 leu2Δ0 his3Δ0 met15Δ0 Cln3Δ103-13Myc-His3MX*	3A
YJB506	*MATa ura3Δ0 leu2Δ0 his3Δ0 met15Δ0 Cln3Δ126-13Myc-His3MX*	3A
YJB494	*MATa his3Δ1 ura3Δ0 leu2Δ0 grr1Δ::NLS-mRFP-LEU2 cdc4-1-Hyg CLN3Δ22-13MYC-HIS3MX +pYES2-GST-URA3*	3B
YJB495	*MATa his3Δ1 ura3Δ0 leu2Δ0 grr1Δ::NLS-mRFP-LEU2 cdc4-1-Hyg CLN3Δ22-13MYC-HIS3MX +pYES2-GST- CDC4ΔF-FLAG-URA3*	3B
YJB496	*MATa his3Δ1 ura3Δ0 leu2Δ0 grr1Δ::NLS-mRFP-LEU2 cdc4-1-Hyg CLN3Δ22-13MYC-HIS3MX +pYES2-GST- GRR1ΔF-FLAG-URA3*	3B
YJB491	*MATa his3Δ1 ura3Δ0 leu2Δ0 grr1Δ::NLS-mRFP-LEU2 cdc4-1-Hyg CLN3Δ103-13MYC-HIS3MX +pYES2-GST-URA3*	3B
YJB492	*MATa his3Δ1 ura3Δ0 leu2Δ0 grr1Δ::NLS-mRFP-LEU2 cdc4-*	3B

	1-Hyg CLN3Δ103-13MYC-HIS3MX +pYES2-GST- CDC4ΔF-FLAG-URA3	
YJB493	*MATa his3Δ1 ura3Δ0 leu2Δ0 grr1Δ::NLS-mRFP-LEU2 cdc4-1-Hyg CLN3Δ103-13MYC-HIS3MX +pYES2-GST- GRR1ΔF-FLAG-URA3*	3B
YBL150	*MATa his3Δ1 ura3Δ0 leu2Δ0 grr1Δ::NLS-mRFP-LEU2 cdc4-1-Hyg CLN3Δ126-13MYC-HIS3MX +pYES2-GST-URA3*	3B
YBL151	*MATa his3Δ1 ura3Δ0 leu2Δ0 grr1Δ::NLS-mRFP-LEU2 cdc4-1-Hyg CLN3Δ126-13MYC-HIS3MX +pYES2-GST- CDC4ΔF-FLAG-URA3*	3B
YBL152	*MATa his3Δ1 ura3Δ0 leu2Δ0 grr1Δ::NLS-mRFP-LEU2 cdc4-1-Hyg CLN3Δ126-13MYC-HIS3MX +pYES2-GST- GRR1ΔF-FLAG-URA3*	3B
YBL147	*MATa his3Δ1 ura3Δ0 leu2Δ0 grr1Δ::NLS-mRFP-LEU2 cdc4-1-Hyg CLN3-1-13MYC-HIS3MX +pYES2-GST-URA3*	3B
YBL148	*MATa his3Δ1 ura3Δ0 leu2Δ0 grr1Δ::NLS-mRFP-LEU2 cdc4-1-Hyg CLN3-1-13MYC-HIS3MX +pYES2-GST- CDC4ΔF-FLAG-URA3*	3B
YBL149	*MATa his3Δ1 ura3Δ0 leu2Δ0 grr1Δ::NLS-mRFP-LEU2 cdc4-1-Hyg CLN3-1-13MYC-HIS3MX +pYES2-GST- GRR1ΔF-FLAG-URA3*	3B
YJB419	*MATa his3Δ1 ura3Δ0 leu2Δ0 CLN3-13MYC-HIS3 grr1Δ::NLS-RFP-LEU2 cdc4-1-Hyg + pYES2-GST-URA3*	3B, S2A, 4A
YJB420	*MATa his3Δ1 ura3Δ0 leu2Δ0 CLN3-13MYC-HIS3 grr1Δ::NLS-RFP-LEU2 cdc4-1-Hyg + pYES2-GST-CDC4-FLAG-URA3*	S2A
YJB421	*MATa his3Δ1 ura3Δ0 leu2Δ0 CLN3-13MYC-HIS3 grr1Δ::NLS-RFP-LEU2 cdc4-1-Hyg + pYES2-GST-CDC4ΔF-FLAG-URA3*	3B, S2A, 4A
YJB422	*MATa his3Δ1 ura3Δ0 leu2Δ0 CLN3-13MYC-HIS3 grr1Δ::NLS-RFP-LEU2 cdc4-1-Hyg + pYES2-GST-GRR1-FLAG-URA3*	S2A
YJB423	*MATa his3Δ1 ura3Δ0 leu2Δ0 CLN3-13MYC-HIS3 grr1Δ::NLS-RFP-LEU2 cdc4-1-Hyg + pYES2-GST-GRR1ΔF-FLAG-URA3*	3B, S2A, 4A
YJB477	*MATα his3Δ1 ura3Δ0 leu2Δ0 lys2Δ grr1::NLS-mRFP-LEU2 cdc4-1-Hyg CLN3-9A-13MYC-HIS3 + pYES2-GST-URA3*	4A
YJB478	*MATα his3Δ1 ura3Δ0 leu2Δ0 lys2Δ grr1::NLS-mRFP-LEU2 cdc4-1-Hyg CLN3-9A-13MYC-HIS3 + pYES2-GST-CDC4ΔF-FLAG-URA3*	4A
YJB479	*MATα his3Δ1 ura3Δ0 leu2Δ0 lys2Δ grr1::NLS-mRFP-LEU2 cdc4-1-Hyg CLN3-9A-13MYC-HIS3 + pYES2-GST-*	4A

	GRR1ΔF-FLAG-URA3	
YJB473-14	*MATa his3Δ1 ura3Δ0 leu2Δ0 lys2Δ0 CLN3-13MYC-HIS3*	4B-D
YJB473-3	*MATa his3Δ1 ura3Δ0 leu2Δ0 lys2Δ0 CLN3-3A-13MYC-HIS3*	4B-D
YJB473-13	*MATa his3Δ1 ura3Δ0 leu2Δ0 lys2Δ0 CLN3-9A-13MYC-HIS3*	4B-C, S3
YJB537	*MATa his3Δ1 ura3Δ0 leu2Δ0 lys2Δ0 CLN3-5A-13MYC-HIS3*	4B-D
YJB538	*MATa his3Δ1 ura3Δ0 leu2Δ0 lys2Δ0 CLN3-5A-13MYC-HIS3 grr1Δ::NLS-RFP-LEU2*	4D
YJB539	*MATα his3Δ1 ura3Δ0 leu2Δ0 lys2Δ0 CLN3-5A-13MYC-HIS3 cdc4-1-Hyg*	4D
YJB535	*MATa his3Δ1 ura3Δ0 leu2Δ0 lys2Δ0 CLN3-3A-13MYC-HIS3 grr1Δ::NLS-RFP-LEU2*	4D
YJB536	*MATα his3Δ1 ura3Δ0 leu2Δ0 lys2Δ0 CLN3-3A-13MYC-HIS3 cdc4-1-Hyg*	4D
YJB296	*MATa his3Δ1 ura3Δ0 leu2Δ0 met15Δ0 HYG-TEFp-CLN3-13MYC-HIS3*	4E
YJB540	*MATa his3Δ1 ura3Δ0 leu2Δ0 met15Δ0 HYG-TEFp-CLN3(207-580)-13MYC-HIS3*	4E
YJB541	*MATa his3Δ1 ura3Δ0 leu2Δ0 met15Δ0 HYG-TEFp-CLN3(207-580)9A-13MYC-HIS3*	4E
YJB407-1	*MATa his3Δ1 ura3Δ0 leu2Δ0 met15Δ0 CLN2-13MYC-HIS3*	5B
YJB408-1	*MATa his3Δ1 ura3Δ0 leu2Δ0 met15Δ0 grr1Δ::NLS-RFP-LEU2 CLN2-13MYC-HIS3*	5B
YJB409-12	*MATα ura3Δ0 leu2Δ0 his3Δ1 lys2Δ0 cdc4-1-Hyg CLN2-13MYC-HIS3*	5B
YJB410-2	*MATa his3Δ1 ura3Δ0 leu2Δ0 grr1Δ::NLS-RFP-LEU2 cdc4-1-Hyg CLN2-13MYC-HIS3*	5B
YJB415-1	*MATa his3Δ1 ura3Δ0 leu2Δ0 met15Δ0 CLN3-2C-13MYC-HIS3*	5B
YJB416-1	*MATa his3Δ1 ura3Δ0 leu2Δ0 met15Δ0 grr1Δ::NLS-RFP-LEU2 CLN3-2C-13MYC-HIS3*	5B
YJB417-3	*MATα ura3Δ0 leu2Δ0 his3Δ1 lys2Δ0 cdc4-1-Hyg CLN3-2C-13MYC-HIS3*	5B
YJB418-3	*MATa his3Δ1 ura3Δ0 leu2Δ0 grr1Δ::NLS-RFP-LEU2 cdc4-1-Hyg CLN3-2C-13MYC-HIS3*	5B
YJB411-3	*MATa his3Δ1 ura3Δ0 leu2Δ0 met15Δ0 CLN2-3C-13MYC-HIS3*	5B
YJB412-2	*MATa his3Δ1 ura3Δ0 leu2Δ0 met15Δ0 grr1Δ::NLS-RFP-LEU2 CLN2-3C-13MYC-HIS3*	5B
YJB413-2	*MATα ura3Δ0 leu2Δ0 his3Δ1 lys2Δ0 cdc4-1-Hyg CLN2-3C-13MYC-HIS3*	5B
YJB435-1	*MATa his3Δ1 ura3Δ0 leu2Δ0 met15Δ0 grr1Δ::NLS-RFP-LEU2 CLN3Δ22-13MYC-HIS3*	5C, S3

YBL100	*MATa his3Δ1 ura3Δ0 leu2Δ0 met15Δ0 grr1Δ::NLS-RFP-LEU2 CLN2-13MYC-HIS3 + pYES2-GST-URA3*	6A-B
YJB480	*MATa his3Δ1 ura3Δ0 leu2Δ0 met15Δ0 grr1Δ::NLS-RFP-LEU2 CLN2-4T3S-13MYC-HIS3 + pYES2-GST-URA3*	6A-B
YJB481	*MATa his3Δ1 ura3Δ0 leu2Δ0 met15Δ0 grr1Δ::NLS-RFP-LEU2 CLN2-13MYC-HIS3 + pYES2-GST-CDC4ΔF-FLAG-URA3*	6A
YJB482	*MATa his3Δ1 ura3Δ0 leu2Δ0 met15Δ0 grr1Δ::NLS-RFP-LEU2 CLN2-4T3S-13MYC-HIS3 + pYES2-GST-CDC4ΔF-FLAG-URA3*	6A
YJB483	*MATa his3Δ1 ura3Δ0 leu2Δ0 met15Δ0 grr1Δ::NLS-RFP-LEU2 CLN2-13MYC-HIS3 + pYES2-GST-GRR1ΔF-FLAG-URA3*	6A
YJB484	*MATa his3Δ1 ura3Δ0 leu2Δ0 met15Δ0 grr1Δ::NLS-RFP-LEU2 CLN2-4T3S-13MYC-HIS3 + pYES2-GST-GRR1ΔF-FLAG-URA3*	6A
YBL101	*MATa his3Δ1 ura3Δ0 leu2Δ0 met15Δ0 grr1Δ::NLS-RFP-LEU2 CLN2-13MYC-HIS3 + pYES2-GST-CDC4-FLAG-URA3*	6B
YBL102	*MATa his3Δ1 ura3Δ0 leu2Δ0 met15Δ0 grr1Δ::NLS-RFP-LEU2 CLN2-13MYC-HIS3 + pYES2-GST-NES-CDC4-FLAG-URA3*	6B
YJB529	*MATa his3Δ1 ura3Δ0 leu2Δ0 met15Δ0 grr1Δ::NLS-RFP-LEU2 CLN2-4T3S-13MYC-HIS3 + pYES2-GST-CDC4-FLAG-URA3*	6B
YJB530	*MATa his3Δ1 ura3Δ0 leu2Δ0 met15Δ0 grr1Δ::NLS-RFP-LEU2 CLN2-4T3S-13MYC-HIS3 + pYES2-GST-NES-CDC4-FLAG-URA3*	6B
YJD95-1	*MATα his3Δ1 ura3Δ0 leu2Δ0 rgt1Δ::KanMX*	7
YJD53-1	*MATα his3Δ1 ura3Δ0 leu2Δ0 rgt1Δ::KanMX KanMX grr1Δ::NLS-RFP-LEU2*	7
YJD93-1	*MATα his3Δ1 ura3Δ0 leu2Δ0 rgt1Δ::KanMX cdc4-1-Hyg*	7
YJD52-1	*MATα his3Δ1 ura3Δ0 leu2Δ0 rgt1Δ::KanMX grr1Δ::NLS-RFP-LEU2 cdc4-1-Hyg*	7
YBL128-1	*MATα his3Δ1 ura3Δ0 leu2Δ0 rgt1Δ::KanMX cln3Δ::HIS3*	7
YBL130-4	*MATα his3Δ1 ura3Δ0 leu2Δ0 rgt1Δ::KanMX KanMX grr1Δ::NLS-RFP-LEU2 cln3Δ::HIS3*	7
YBL132-8	*MATα his3Δ1 ura3Δ0 leu2Δ0 rgt1Δ::KanMX cdc4-1-Hyg cln3Δ::HIS3*	7
YBL134-7	*MATα his3Δ1 ura3Δ0 leu2Δ0 rgt1Δ::KanMX grr1Δ::NLS-*	7

	RFP-LEU2 cdc4-1-Hyg cln3Δ::HIS3	
YJB486	*MATa ura3Δ0 leu2Δ0 his3Δ0 met15Δ0 CLN1-13MYC-HIS3Mx*	S4
YJB487	*MATa ura3Δ0 leu2Δ0 his3Δ0 met15Δ0 0 grr1Δ::NLS-mRFP-LEU2 CLN1-13MYC-HIS3Mx*	S4
YJB507	*MATa ura3Δ0 leu2Δ0 his3Δ0 met15Δ0 0 grr1Δ::NLS-mRFP-LEU2 CLN1-13MYC-HIS3Mx +pYES2-GST-URA3*	S4
YJB508	*MATa ura3Δ0 leu2Δ0 his3Δ0 met15Δ0 0 grr1Δ::NLS-mRFP-LEU2 CLN1-13MYC-HIS3Mx +pYES2-GST- CDC4ΔF-FLAG-URA3*	S4
YJB509	*MATa ura3Δ0 leu2Δ0 his3Δ0 met15Δ0 0 grr1Δ::NLS-mRFP-LEU2 CLN1-13MYC-HIS3Mx +pYES2-GST- GRR1ΔF-FLAG-URA3*	S4
